# Supplementary material for: Macromolecule conformational shaping for extreme mechanical programming of polymorphic hydrogel fibers
Source: Nat Commun. 2022 Jun 11;13:3369. doi: 10.1038/s41467-022-31047-3 (PMC9188594; doi:10.1038/s41467-022-31047-3)
Supplement: Supplementary file 1 — Supplementary Information [file 41467_2022_31047_MOESM1_ESM.pdf]

Supplementary Information for

Macromolecule conformational shaping for extreme mechanical programming of polymorphic hydrogel fibers

Xiao-Qiao Wang[1], Kwok Hoe Chan[1], Wanheng Lu[1], Tianpeng Ding[1], Serene Wen Ling Ng[1], Yin Cheng[1], Tongtao Li[1], Minghui Hong[1], Benjamin C. K. Tee[1,2], Ghim Wei Ho[1,2]*

Dr. X. Q. Wang, K. H. Chan, Dr. W. Lu, Dr. T. P. Ding, Dr. S. W. L. Ng, Dr. Y. Cheng, Dr. T. T. Li, Prof. M. Hong, Prof. B. C. K. Tee, Prof. G. W. Ho
Department of Electrical and Computer Engineering
National University of Singapore
4 Engineering Drive 3, Singapore 117583, Singapore

Prof. B. C. K. Tee
Prof. G. W. Ho
Department of Materials Science and Engineering
National University of Singapore,
9 Engineering Drive 1, 117575, Singapore
*Correspondence: elehgw@nus.edu.sg

Supplementary Methods

Design of the robotic bird with installed hydrogel fiber sensor, bluetooth chip and power source. The commercial bionic bird called Metafly was bought and reworked. A lightweight pH 12.38 hydrogel microfiber was installed between one wing and the head. A low-dropout regulator (NCV8161) was used to provide a regulated voltage of 3.3 V to the microcontroller (ESP32). An on board lithium polymer battery also powers the miniature motor that flaps the bird's wing. The regulated 3.3 V is also fed to a potential divider to derive the strain response signal against a resistive load of 100 kΩ. The electrical signal was sampled at 200 Hz using the onboard ADC on the ESP32 microcontroller and wirelessly transmitted to a mobile phone via Bluetooth. A mobile application was developed to acquire, store and chart the incoming response signal, allowing users to monitor the strain response remotely.

Characterization and Measurement. pH values of the hydrogel dopes were measured by the Orion 3-Star Benchtop pH Meter. A MCR302 rheometer (Anton Paar) with cone-plate geometry was used for tests at 21 °C. Viscometry measurements were carried out over shear rates ranging from 0.01 to 100 s^{-1}. Oscillatory amplitude sweeps were performed at 1 Hz within strain values from 0.01% to 1000%. The SEM characterization of the dried hydrogel dopes was accomplished using field emission scanning electron microscopy (FESEM, JEOL FEG JSM 7001F). The filamentation process of polyelectrolyte hydrogels in the methanol bath was captured by an S-EYE Setup Microscope Camera. AFM images were collected on a commercial scanning probe microscope (SPM) instrument (MFP-3D, Asylum Research, CA, USA). Optical microscope images of the hydrogel fibers were captured by an Olympus BX53M microscope in polarization and transmission modes. In the polarization mode, the analyzer and polarizer were oriented perpendicular to each other, the fibers placed between two polarizers were oriented at a 45 degree angle to both the polarizer and analyzer. Confocal microscope images were collected by Nikon C2 microscope. The 3D surface profiles and surface roughness of the hydrogel fibers of different pH were measured and analyzed by an OLS5000 laser-scanning microscope. Small-angle X-ray scattering (SAXS) measurements were performed on a Xenocs Xeuss 2.0 system with a Cu Kα radiation of 1.5418 Å by mounting the hydrogel microfibers perpendicular with respect to the X-ray beam. Wide-angle X-ray scattering (WAXS) measurements were performed using a SWAXS Xenocs Nanoinxider with a Cu Kα radiation of 1.5418 Å and beam path in vacuum. The tensile strain-stress tests were implemented using the tensile machine (MultiTest 1-i) with a 5-N load cell: The length of hydrogel fiber specimens was fixed at 20 mm, and stretched/released at a constant strain rate of 2 min^{-1} (40 mm min^{-1}) unless otherwise stated. The elastic moduli were determined by the initial slopes of the stress-strain curves. The toughness was calculated by integrating the area under the strain-stress curve before the fiber fracture. The mechanical resilience (Re) was calculated:

$$Re = 1 - \frac{\oint s\mathrm{d}\varepsilon}{\int_0^{\varepsilon_{\max}} s\mathrm{d}\varepsilon},$$

where $\oint s\mathrm{d}\varepsilon$ is the area of hysteresis loop in the loading-unloading cycle, $\int_0^{\varepsilon_{\max}} s\mathrm{d}\varepsilon$ the area under the loading curve, s the tensile stress, and $\varepsilon_{\max}$ the maximum tensile strain in the cycle.

The strain recovery was determined by the ratio of the residual strain to the applied maximum

strain. The electric conductivity was calculated based on the equation:

$\sigma = L/(S \times R)$,

where L, S, σ, and R are the length, cross-sectional area, conductivity, and electrical resistance of the hydrogel fiber, respectively. Spring indices of the Janus hydrogel fibers were determined by the ratio of the spring diameter to the Janus fiber thickness. The resistance change of the fibers was measured by the multimeter Keithley DMM6500. The open-circuit voltage and short-circuit current of the hydrogel devices were respectively measured and recorded by the nanovoltmeter (Keithley 2182 A) and electrometer (Keithley 6517B). The infrared images and temperatures were captured and analyzed using the FLIR E50 infrared camera.

Supplementary Figures

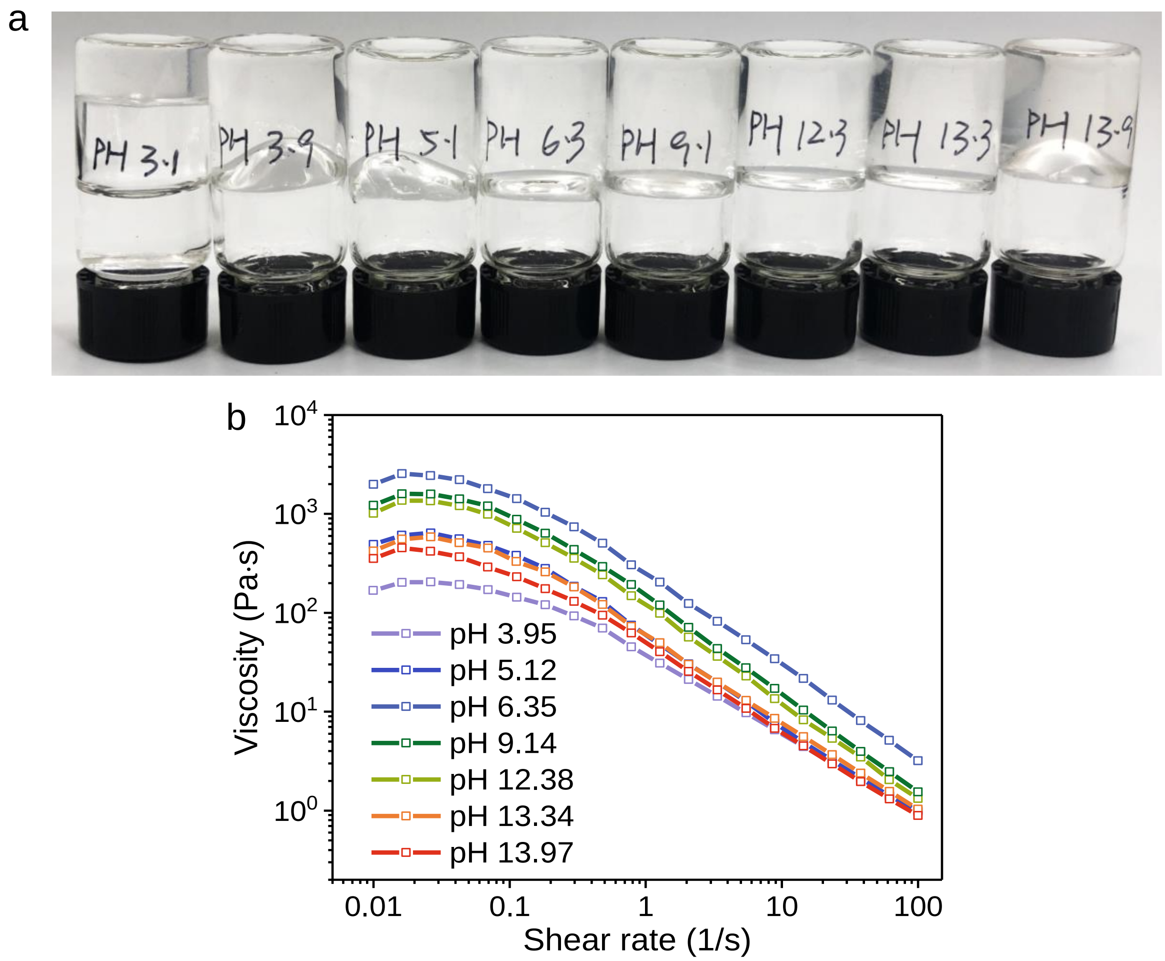

Supplementary Figure 1. Polyelectrolyte hydrogel dopes of different pH. (a) Photograph of the hydrogel dopes. The content of PANa in the dope solutions was fixed at 3.5 wt%. (**b)** Viscosity of the dopes as a function of shear rate.

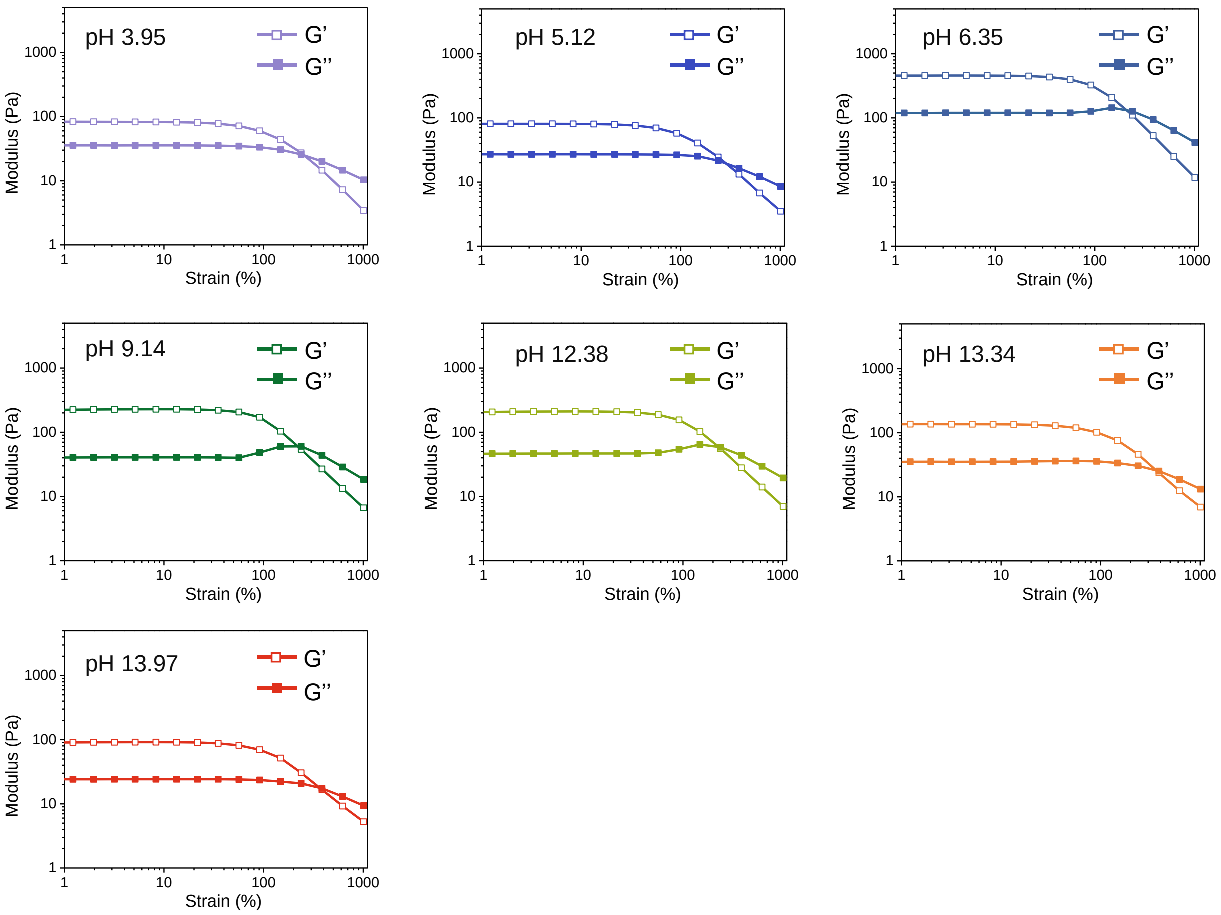

Supplementary Figure 2. Strain-dependent oscillatory rheology of the polyelectrolyte hydrogel dopes of different pH.

At low pH of 3.95 and 5.12, considerable carboxylate groups are protonated, and the uncharged macromolecules assume distributed, random coil conformations with bad affinity with water molecules, resulting in low viscosity; in the moderate pH range, the majority of carboxylate groups are negatively charged, the macromolecules adopt extended, entangled state, and the continuously interpenetrated macromolecule network uniformly bonds with water molecules, leading to high viscosity and modulus; in the high pH range of 13 to 14, the low viscosity can be ascribed to the enhanced interaction of extended macromolecule chains at high NaOH concentration that results in decrease of the overall mutual entanglement of polymer networks. This explanation is further supported by SEM results of the dried hydrogel dopes and the structural characterization of the formed hydrogel micofibers.

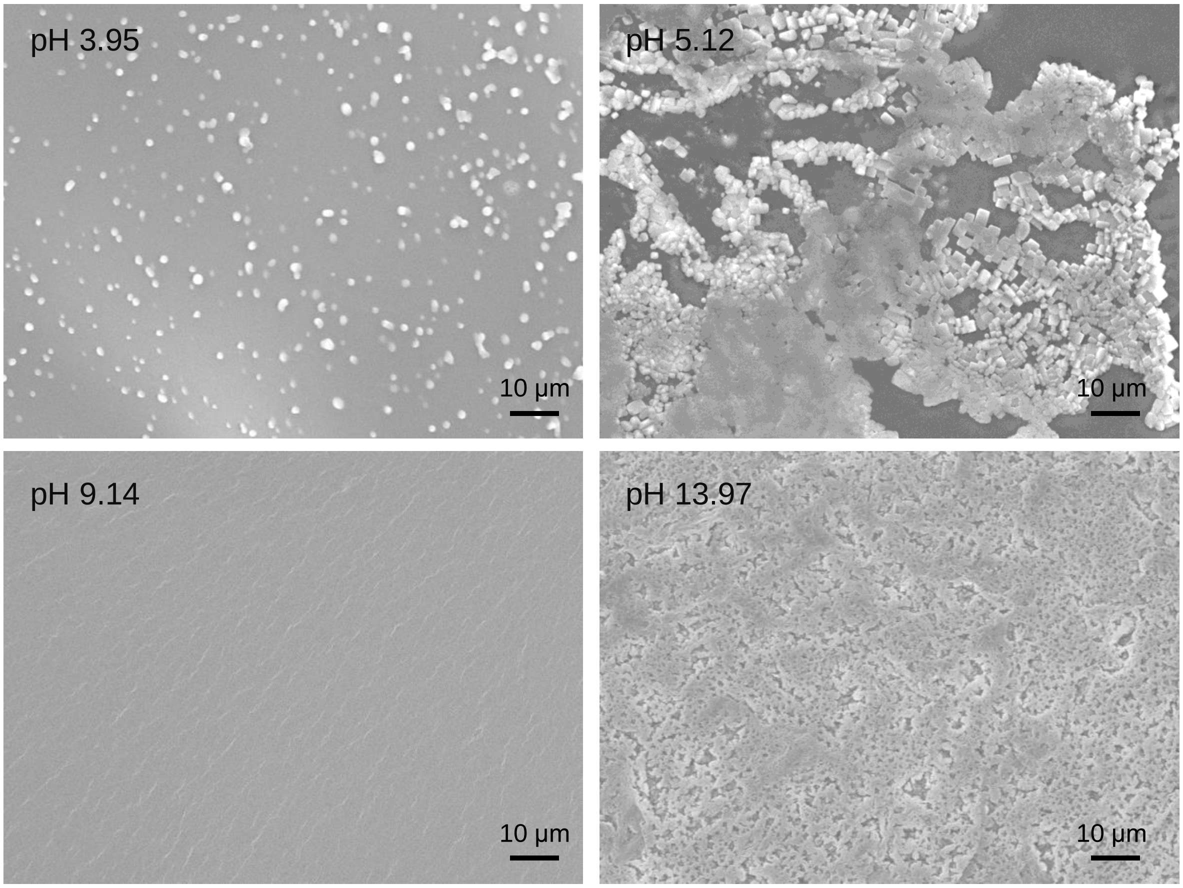

Supplementary Figure 3. SEM images of the dried polyelectrolyte hydrogel dopes.

SEM images of the dried dopes show the presence of aggregated polymer microparticles at pH 3.95 and 5.12, a uniform polymer film at pH 9.14 and polymer phase separation at pH 13.97.

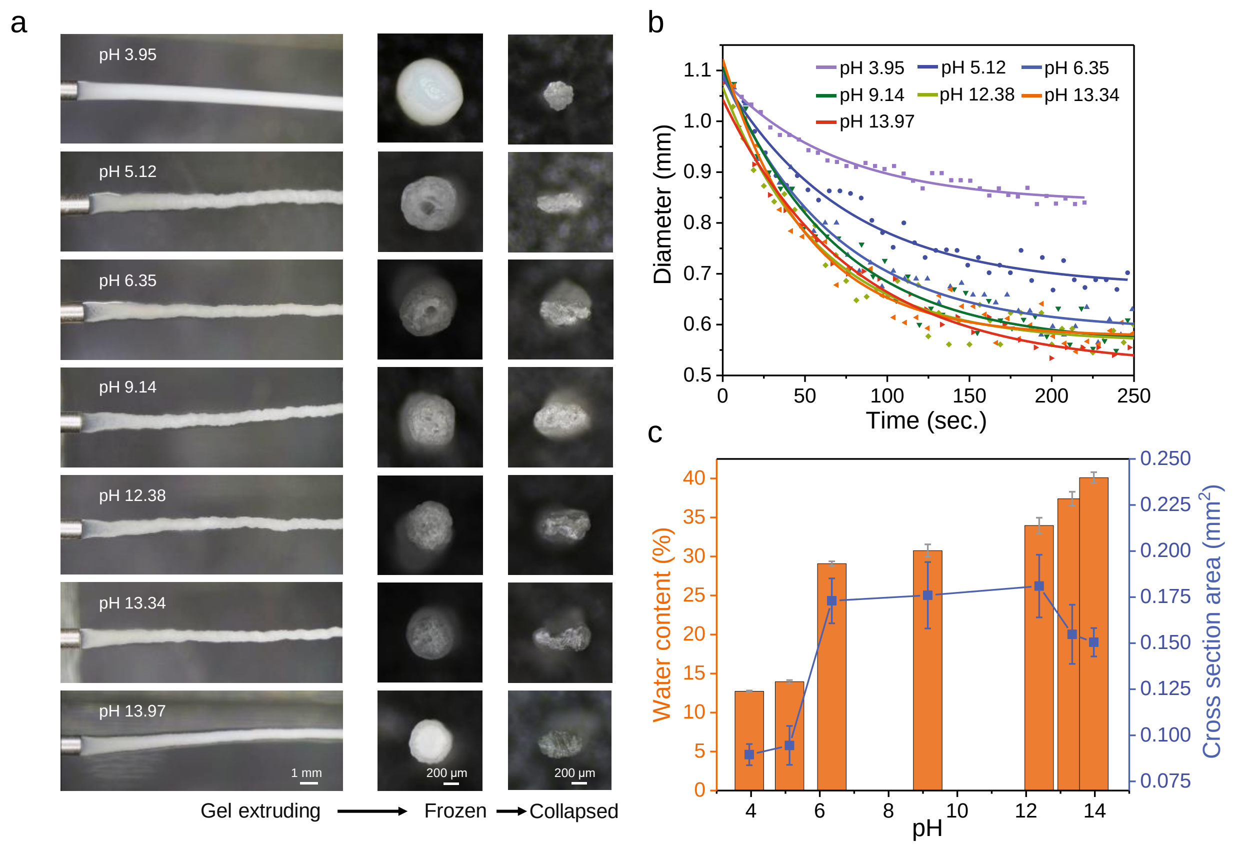

Supplementary Figure 4. Fabrication of polyelectrolyte hydrogel microfibers of different pH. (a) Optical microscope images showing hydrogel filamentation of different pH in methanol, the solidified structures in methanol and finally stabilized structures of the hydrogel microfibers in the ambient air. **(b)** Time-dependent diameter change of the microfibers during filamentation process. **(c)** Equilibrated water contents and cross-sectional areas of the stabilized hydrogel microfibers. The inset figure shows the open-circuit voltage as a function of temperature difference. Error bars represent SD.

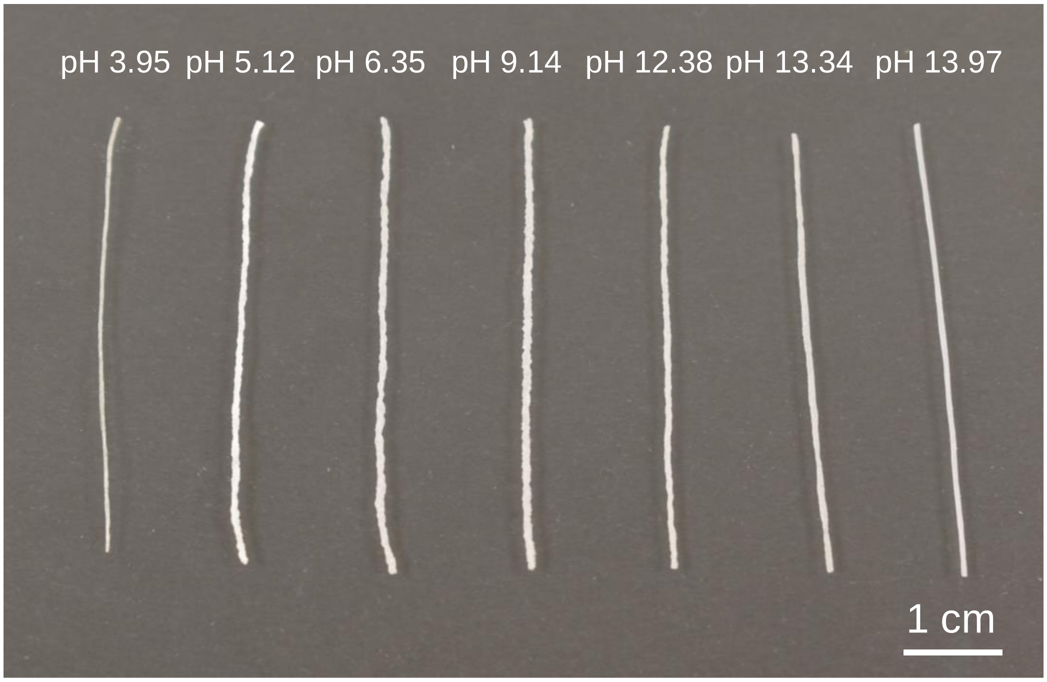

Supplementary Figure 5. Photograph of hydrogel microfibers prepared from dopes of different pH.

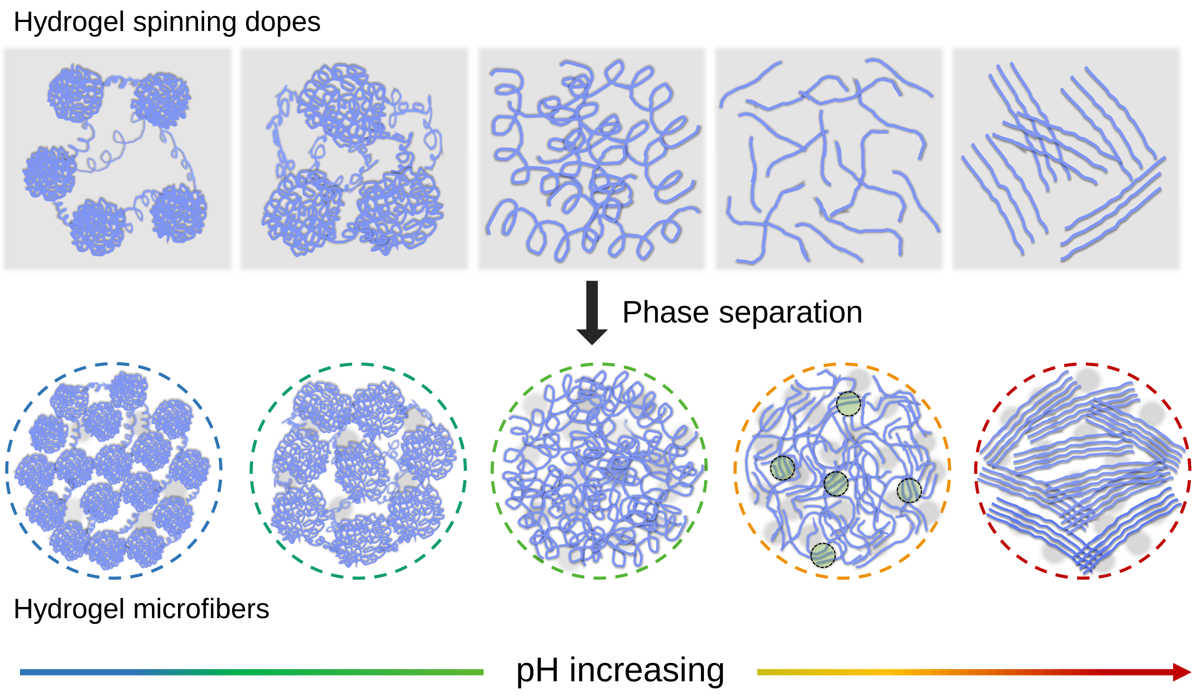

Supplementary Figure 6. Polymer networks of the hydrogel microfibers with macromolecule conformations evolving from tightly coiled to extended, aligned states as pH increases from 3.95 to 13.97.

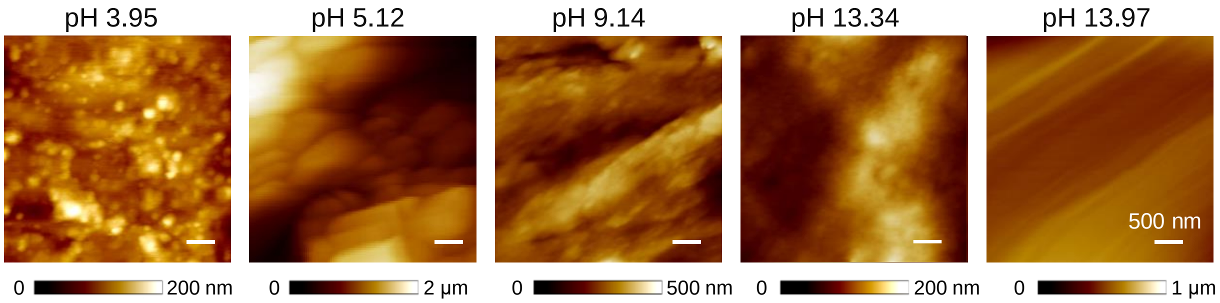

Supplementary Figure 7. AFM images of the hydrogel microfibers of different pH.

AFM images present the nanoscale topography of the hydrogel fibers, where abundant macromolecule globules with particle sizes of tens of nanometers to two hundred nanometers can be observed in the pH 3.95 fiber, and compactly stacked particles of much increased sizes are shown in the pH 5.12 fiber; in the pH 9.14 fiber, we can still observe tiny clusters due to the random entanglement of extended coils in the interpenetrated macromolecule network, while the pH 13.34 fiber reveals fine fibrous structures derived from the extended macromolecule chains; the aggregation of extended macromolecules at pH 13.97 produces clearly aligned textures.

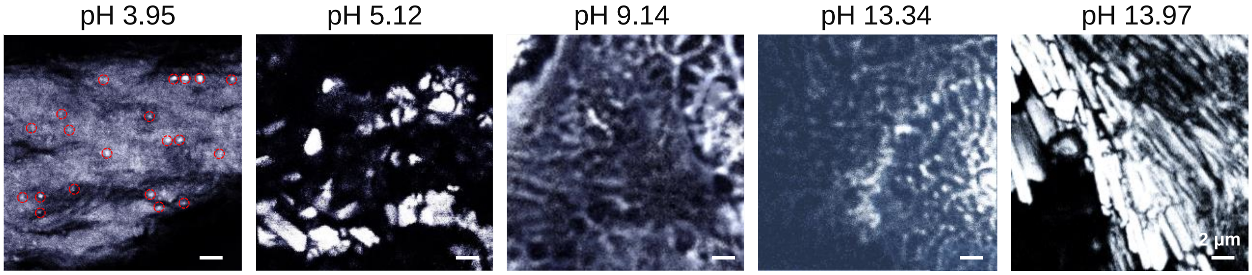

Supplementary Figure 8. Confocal microscope images of hydrogel microfibers.

Confocal microscope images reveal microsized features where abundant globules with particle sizes of hundreds of nanometers are distributed in the pH 3.95 hydrogel fiber; irregular microparticles are observed in the pH 5.12 hydrogel fiber; continuous, porous macromolecule networks are present in the pH 9.14 and pH 13.34 hydrogel fibers; the pH 13.97 hydrogel fiber exhibits compactly aligned polymer fiber bundles.

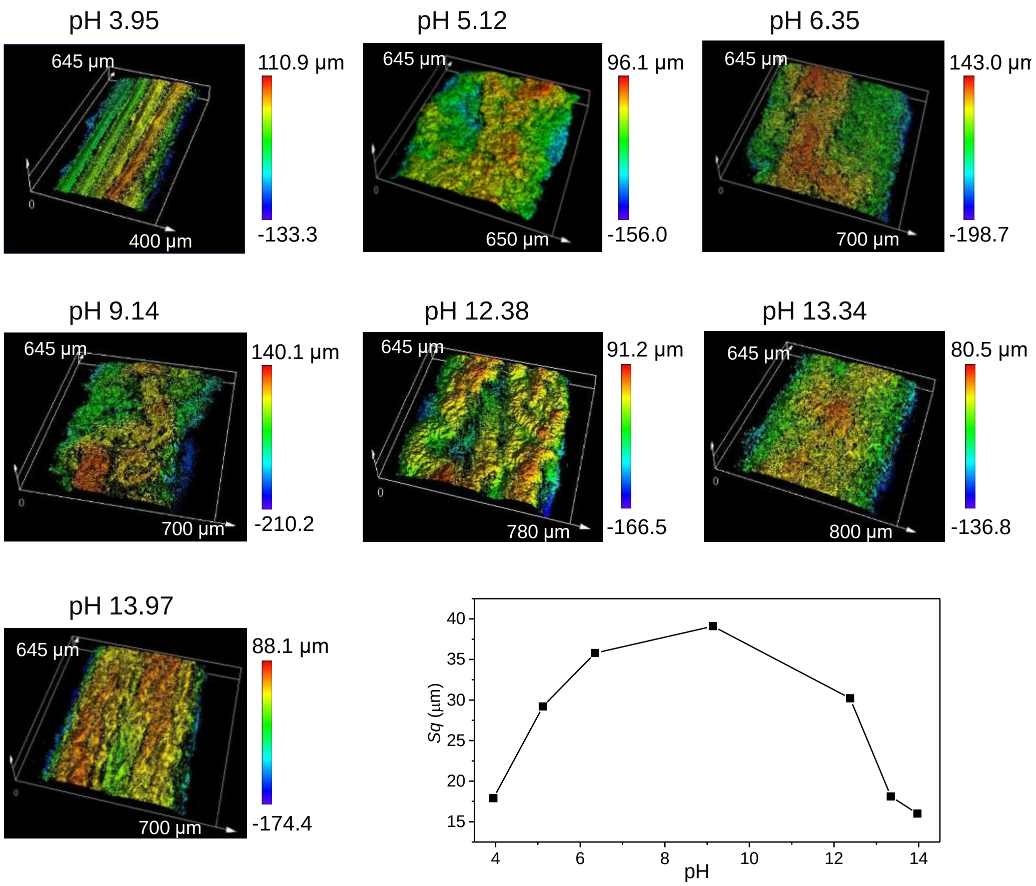

Supplementary Figure 9. The laser-scanning microscopic images of hydrogel microfibers of different pH and the corresponding areal surface roughness.

Hydrogel fibers in the moderate pH range (pH 6.35, 9.14 and 12.38) composed of entangled macromolecule networks exhibit high areal surface roughness (S_q), and hydrogel fibers in the high or low pH range consisting of aggregated macromolecule coils or aligned macromolecules have relatively smoother surfaces.

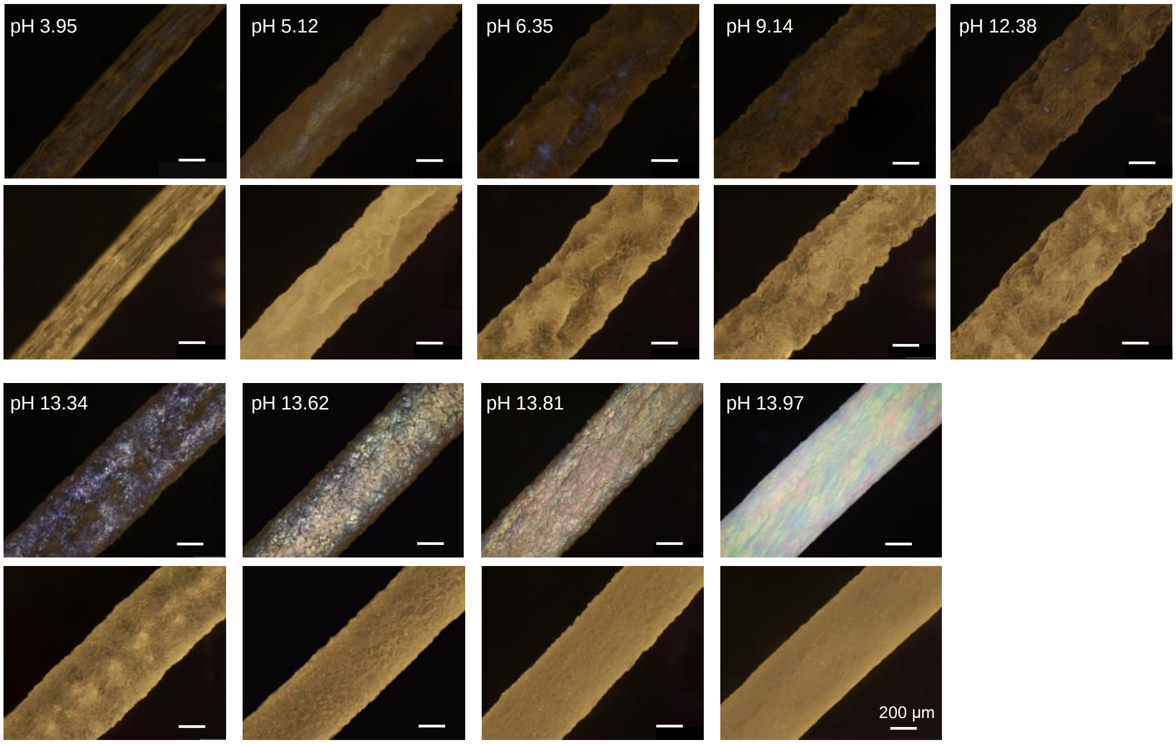

Supplementary Figure 10. Optical microscope images of the hydrogel microfibers in polarization and transmission modes, showing the evolving interference colors and microstructures as pH increase.

Hydrogel fibers in the low to moderate pH range (3.95 to 12.38) show weak interference colors; strong interference colors are revealed in hydrogel fibers of pH 13 to 14, and the sequent color changes from blue, yellow, pink to green, suggesting the increase of birefringence due to the enhancement in macromolecule alignment. The interference color at pH 13.34 is not continuous, indicating that the polyelectrolyte macromolecules in the networks are partially aligned.

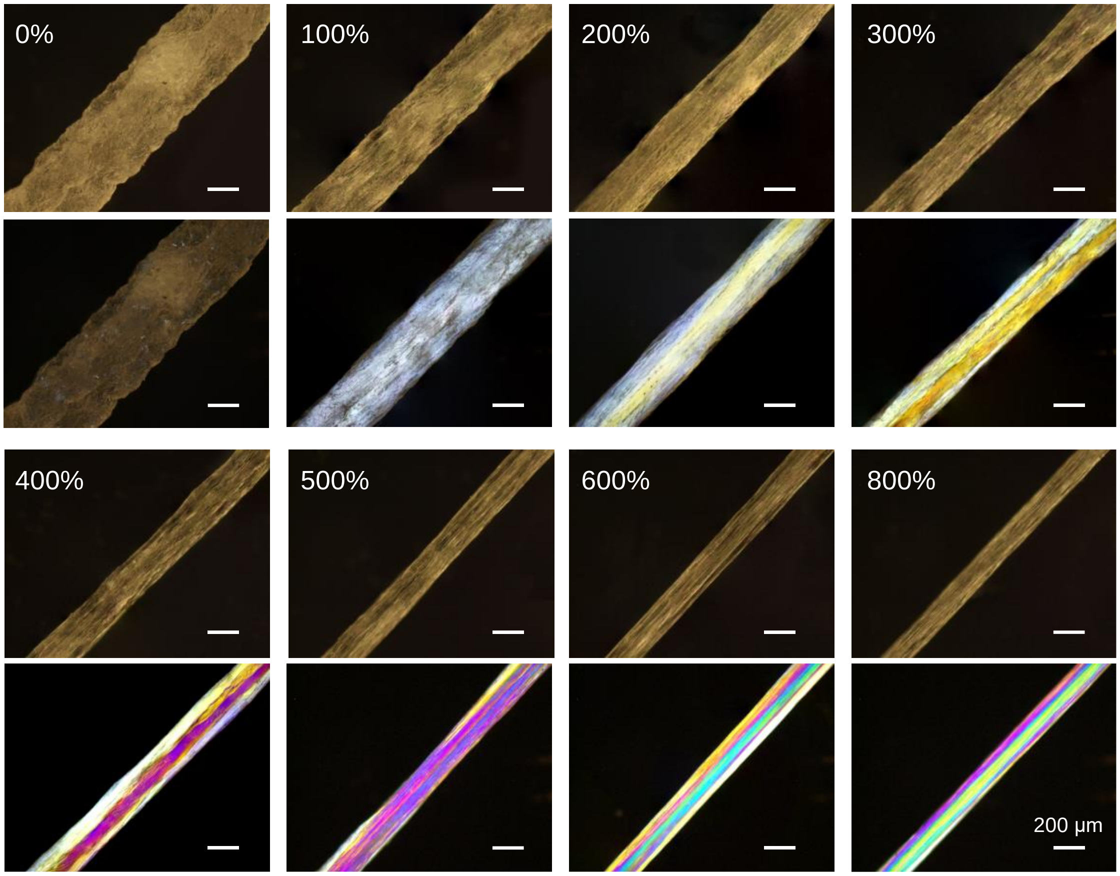

Supplementary Figure 11. Optical microscope images of the pH 9.14 hydrogel microfiber in polarization and transmission modes, showing the evolving interference colors and microstructures as the applied strain gradually increases from 0 to 800%.

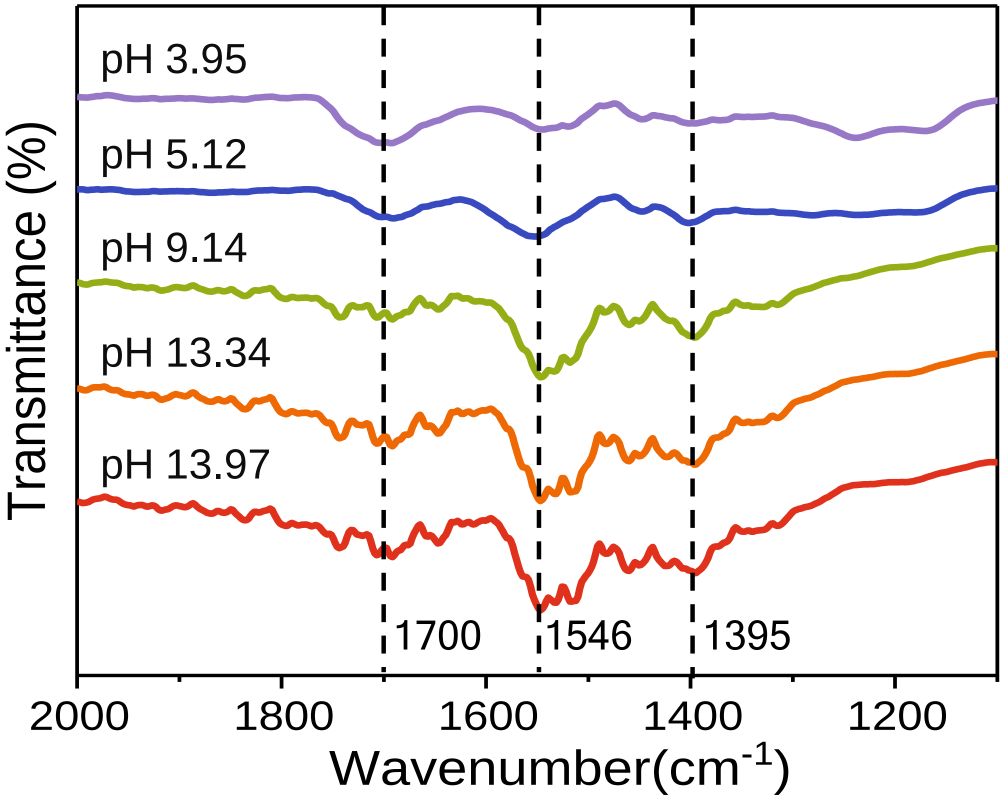

Supplementary Figure 12. Infrared spectra of the dried hydrogel microfibers.

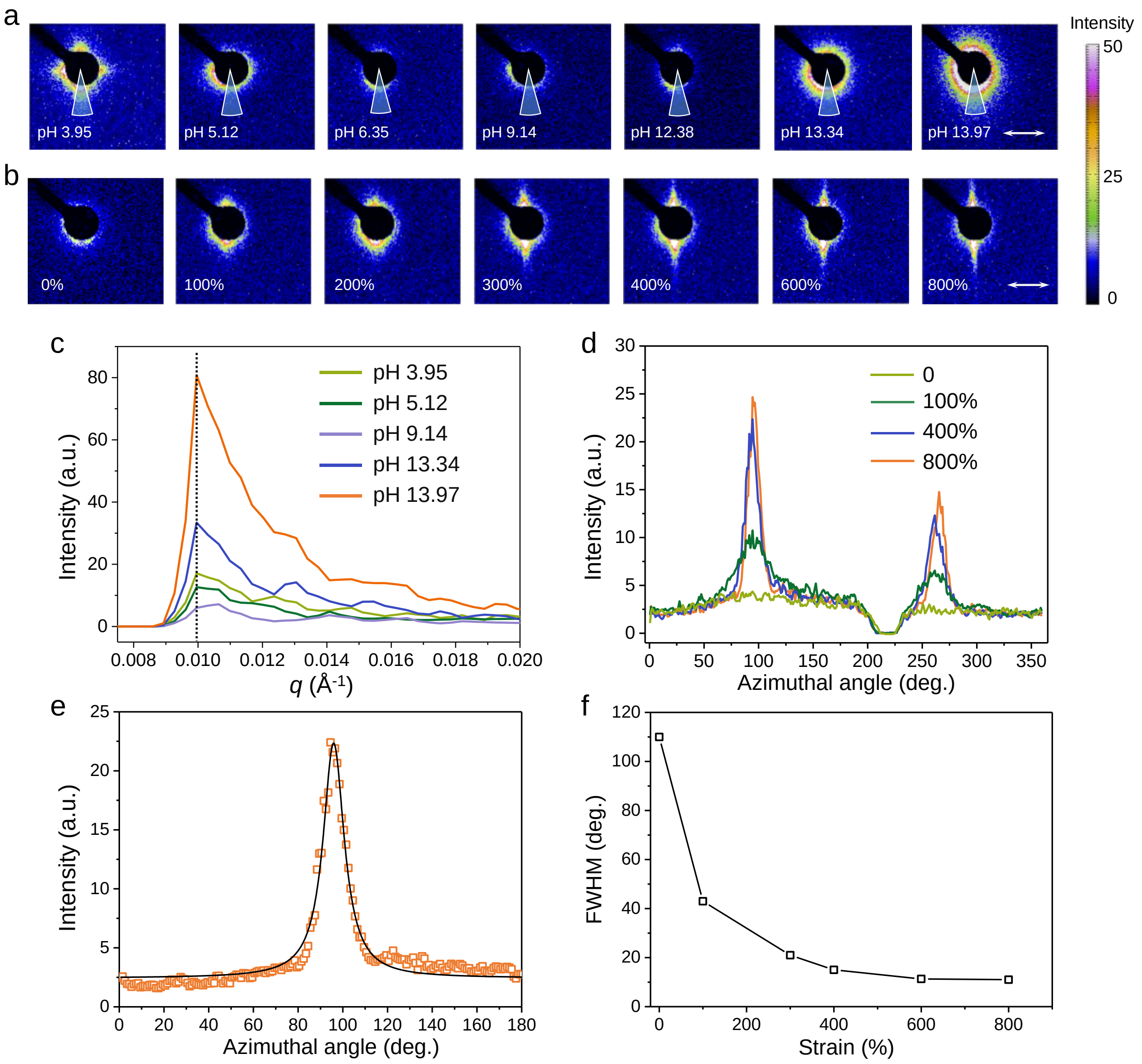

Supplementary Figure 13. Investigation of the delicate structures evolution below 100 nm using SAXS. (a) SAXS patterns and **(c)** profiles of the hydrogel microfibers of different pH. **(b)** SAXS patterns and **(d)** azimuthal angle dependent scattering profiles of pH 9.14 microfiber at different strains. The white double arrows indicate the length direction of the microfibers and the gray sectors indicate the 20° region for intensity integration. **(e)** 1D integrated intensity curve of the pH 9.14 microfiber at 800% strain as a function of the azimuthal angle. Lorentz fitting is used to fit the peak and to obtain the full width at half maximum (FWHM). **(f)** FWHM as a function of strain.

Hydrogel microfibers in the moderate pH range (pH 6.35, 9.14 and 12.38) display very weak

scattering, as the uniformly entangled and continuous networks have low scattering contrast, whereas the hydrogel microfibers (pH 3.95, 13.34 and 13.97) containing aggregated nanoscale domains (polymer nanoclusters or crystalline domains) display high scattering intensity. The strongest diffraction peak at $q = 0.01$ Å^{-1} in the SAXS spectra suggests the presence of aggregated domains with a mean interdomain spacing of ~63 nm (Supplementary Fig. 13a,c). It should be noted that the lab-source SAXS instrument can only detect the signals in the polymer networks where relatively high scattering contrasts are present. Meanwhile, the stretched SAXS pattern of pH 13.97 microfiber indicates the strong scattering perpendicular to the microfiber axis, which is attributed to the aligned nanostructures as shown by the AFM results.

Upon being stretched, the scattering intensity of pH 9.14 hydrogel microfiber becomes stronger perpendicular to the stretching direction, suggesting the appearance of scattering centers induced by the macromolecule reorientation (Supplementary Fig. 13b). We computed FWHM from the azimuthal scan profile to characterize the macromolecule orientation under different strains (Supplementary Fig. 13d-f). FWHM = 180° means an isotropic distribution of the nanonetworks, while FWHM = 0° means a perfectly anisotropic distribution[1]. FWHM is ~110° at 0% strain, and decreases to ~43° at 100% and ~11° at 800% strain.

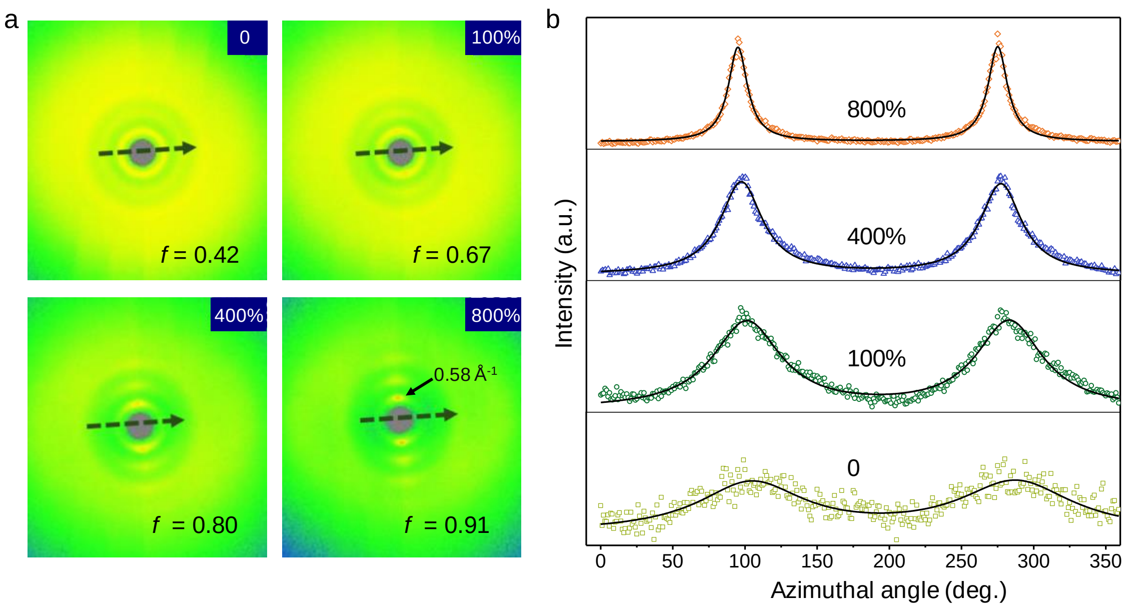

Supplementary Figure 14. Orientation factors of the pH 9.14 microfiber at different strains. (a) WAXS patterns of the microfiber at different strains. The grey arrows indicate the length direction of the microfiber. **(b)** Azimuthal scanning at $q = 0.58$ $Å^{-1}$ and Lorentz fitting results.

Stretching of the pH 9.14 microfiber induces extension and reorientation of the macromolecule chains. Before stretching, a nearly homogenous diffraction ring is observed in the WAXS pattern of the microfiber (Supplementary Fig. 14a). The stretching promotes macromolecule orientation along the microfiber direction, which yields a strong angle dependence in its diffraction pattern with high-intensity arc areas perpendicular to the microfiber axis at 100% and 400% strain. High-intensity diffraction spots are shown in the microfiber at 800% strain. The corresponding integrated intensity scans as a function of the azimuthal angle are shown in Supplementary Fig. 14b. The orientation factor, f is determined by the following equation:

$$f = \frac{180^{\circ} - \Delta\emptyset_{1/2}}{180^{\circ}}$$

where $\Delta\emptyset_{1/2}$ represents FWHM of the azimuthally scanned peak[2]. The value of f is 1 when the polymer chains align perfectly parallel to the fiber axis, and when f is zero, it means there is random orientation. The microfiber at 0% strain gives $f = 0.42$, and $f = 0.91$ at 800% strain indicates that the macromolecule chains are oriented along the microfiber direction.

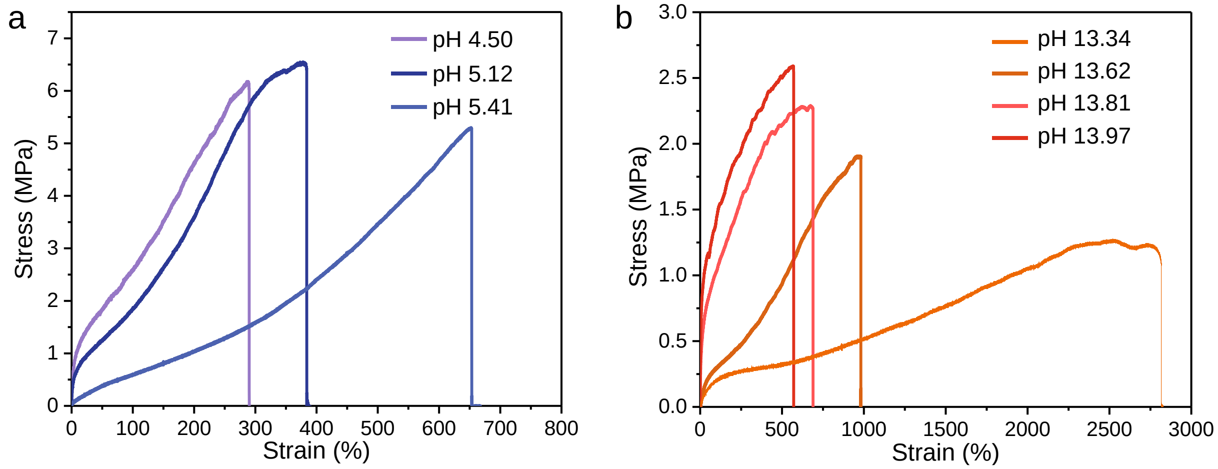

Supplementary Figure 15. (a) Strain-stress curves of the hydrogel microfibers at pH 4.50, 5.12 and 5.41 and (b) pH 13 to 14.

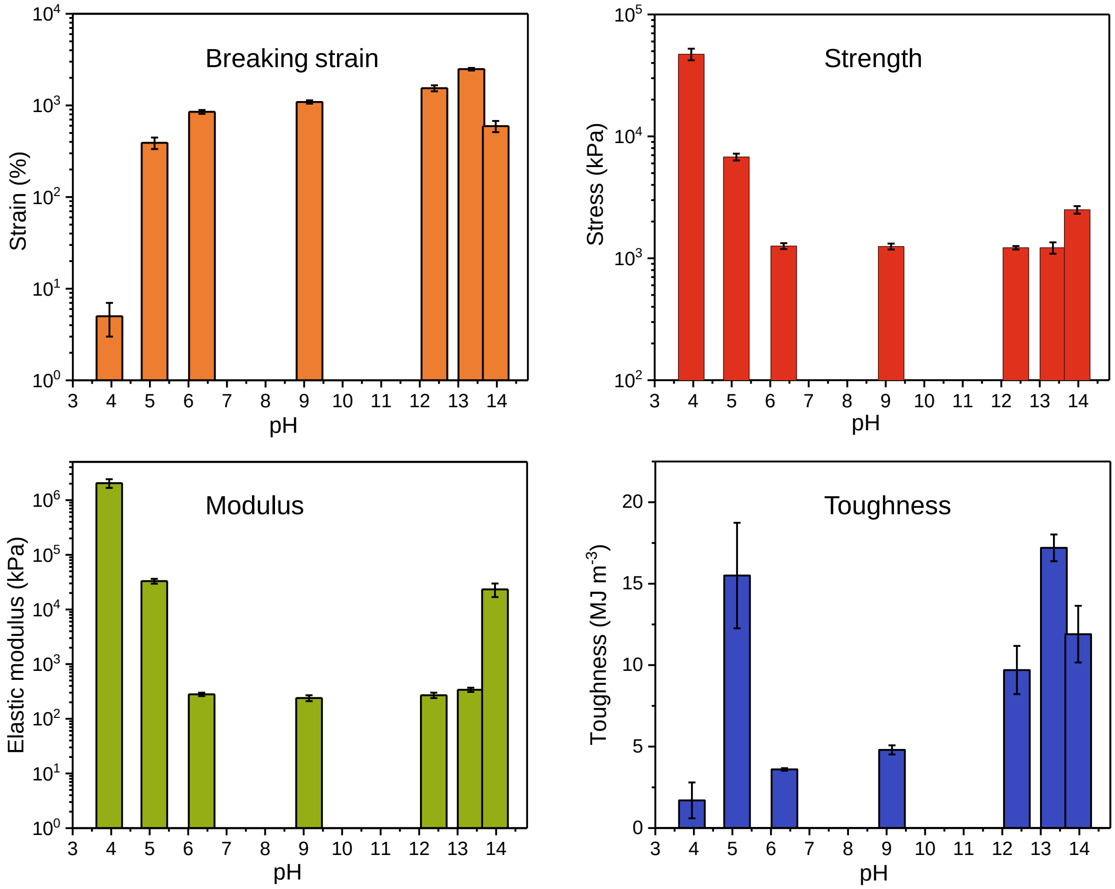

Supplementary Figure 16. Breaking strain, strength, elastic modulus and toughness of the hydrogel microfibers as a function of pH. Error bars represent SD.

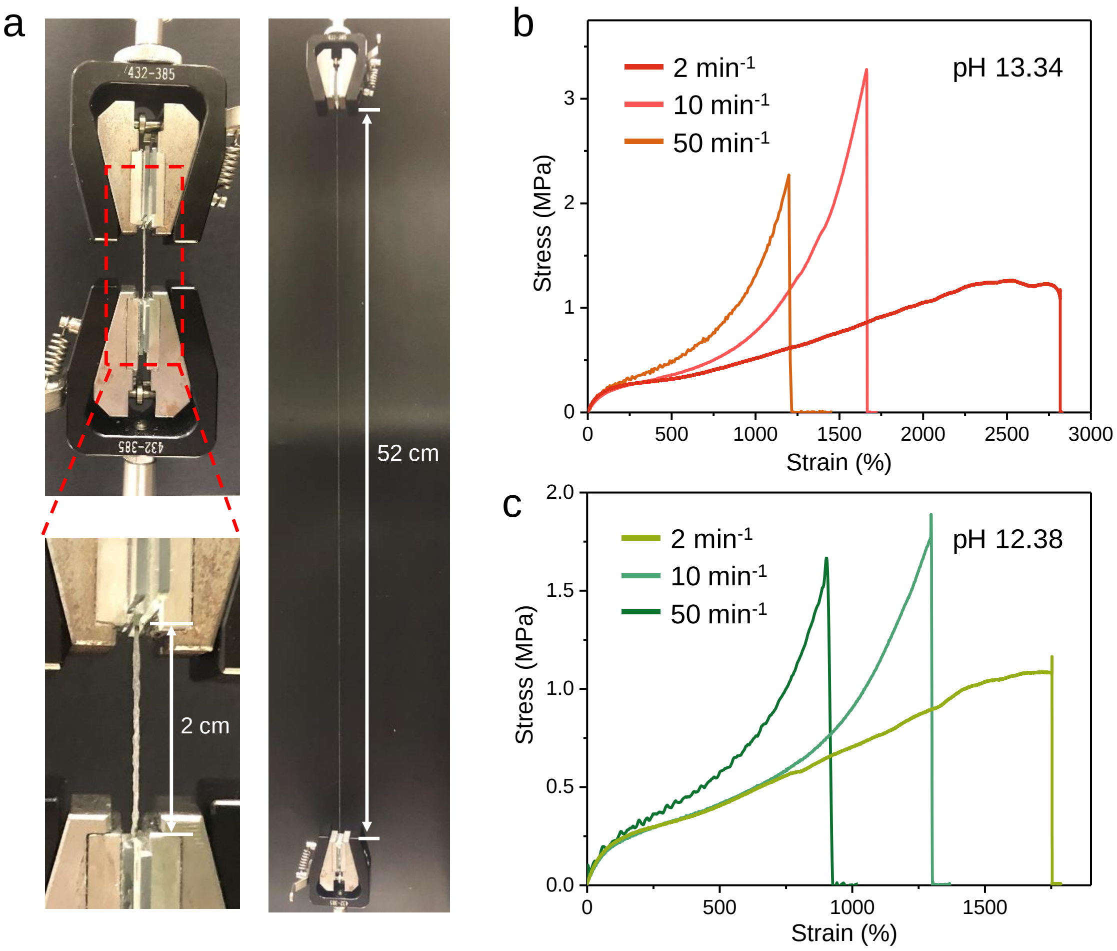

Supplementary Figure 17. Strain rate dependent strain-stress properties of the hydrogel microfibers. (a) Photographs of a pH 13.34 hydrogel microfiber stretched to 2500% strain at a strain rate of 2 min^{-1}. (**b**) Strain rate dependent strain-stress curves of the pH 13.34 and **(c)** pH 12.38 hydrogel microfibers.

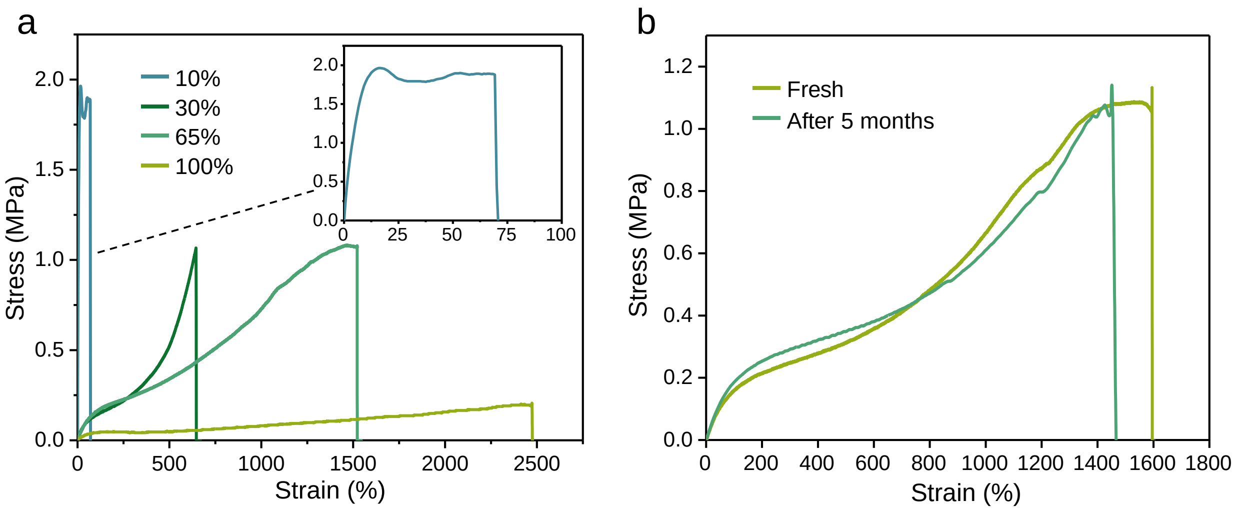

Supplementary Figure 18. (a) Strain-stress curves of the fresh pH 12.38 hydrogel microfiber stabilized at different humidity conditions and (b) the pH 12.38 hydrogel microfiber stored in the ambient environment (65% humidity) for 5 months.

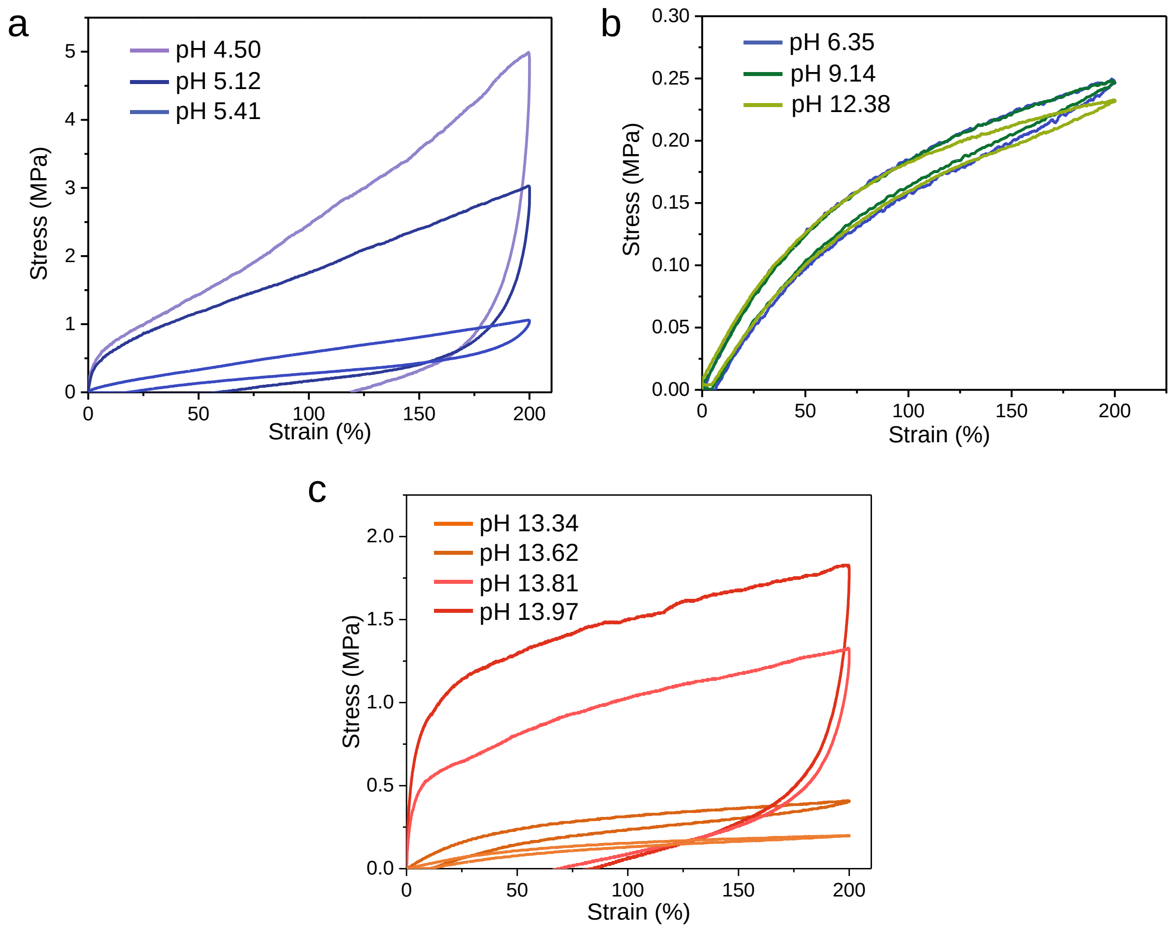

Supplementary Figure 19. Mechanical resilience of hydrogel microfibers of different pH. (a) 200% cyclic strain to the hydrogel microfibers of the low, **(b)** moderate and **(c)** high pH range.

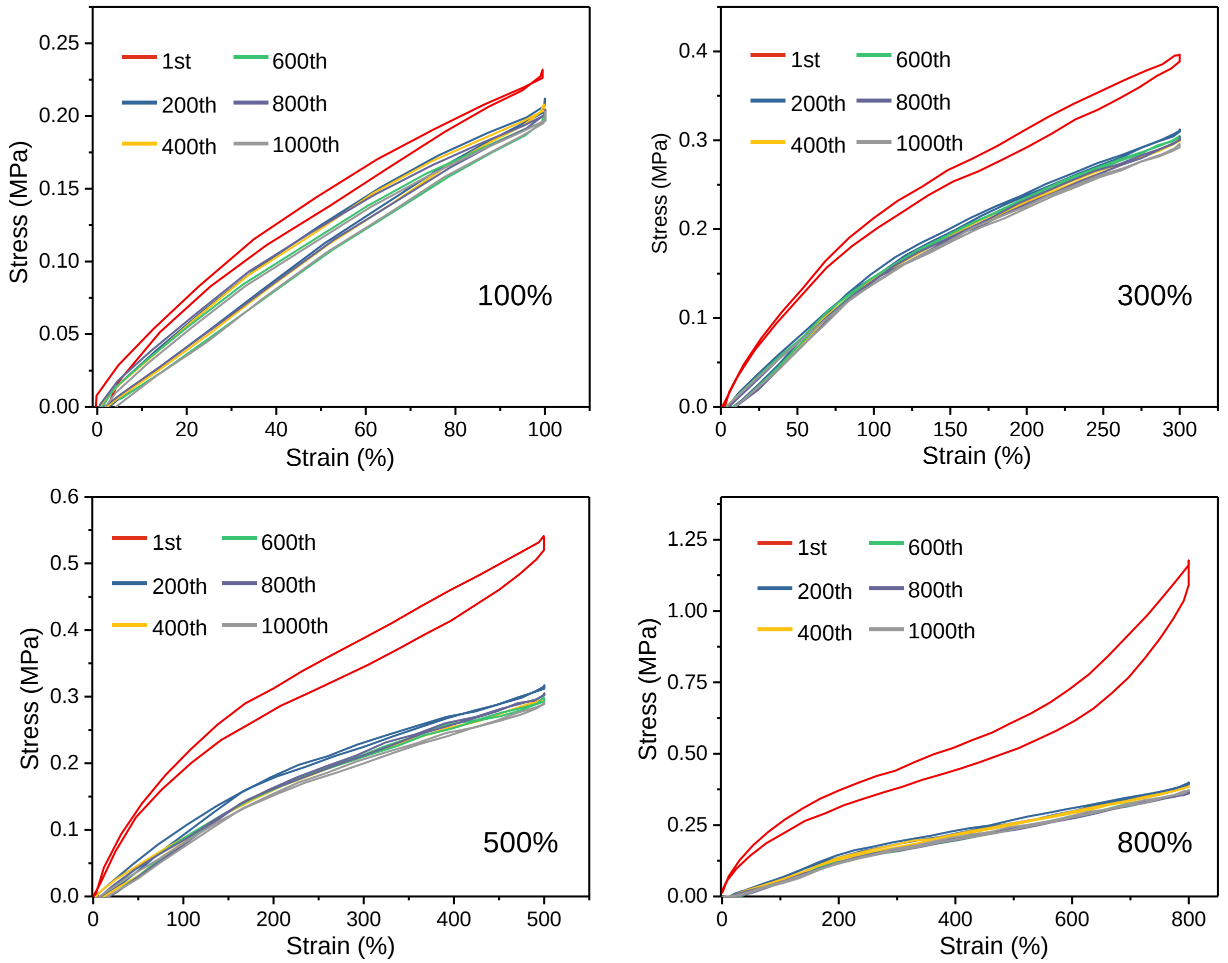

Supplementary Figure 20. Cyclic loading of the pH 12.38 microfiber to different strains for 1000 cycles (strain rate: 50 min^{-1}; waiting time: 10 min for every 100 continuous cycles).

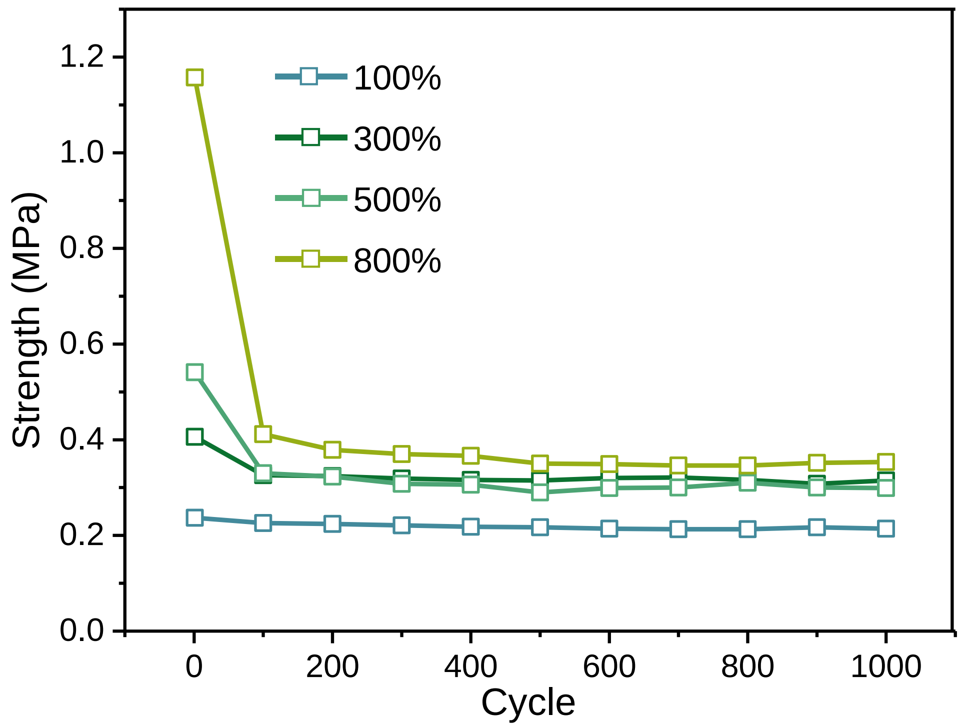

Supplementary Figure 21. Maximum stress of the pH 12.38 hydrogel microfiber at different strains during 1000 cycles (strain rate: 50 min^{-1}; waiting time: 10 min for every 100 continuous cycles).

Maximum stress of the pH 12.38 microfiber subjected to cycled strains of 100%, 300%, 500% and 800% can be stabilized at around 0.21, 0.30, 0.31 and 0.35 MPa, respectively.

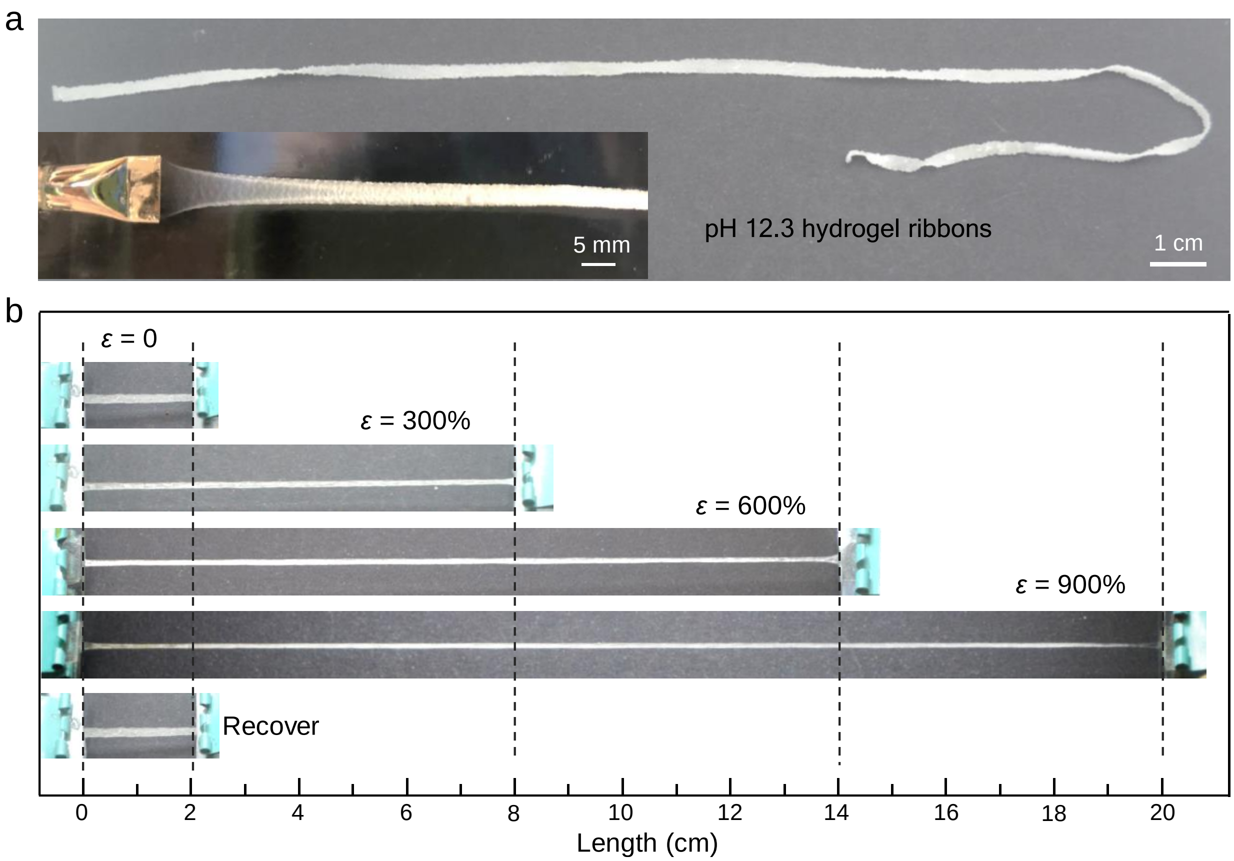

Supplementary Figure 22. Highly stretchable and fast resilient pH 12.38 hydrogel ribbons. (a) Photographs showing the formation of hydrogel ribbon in methanol bath by utilizing a flat nozzle, and the stabilized hydrogel ribbon in the ambient air. **(b)** Stretching and recovery of the hydrogel ribbon.

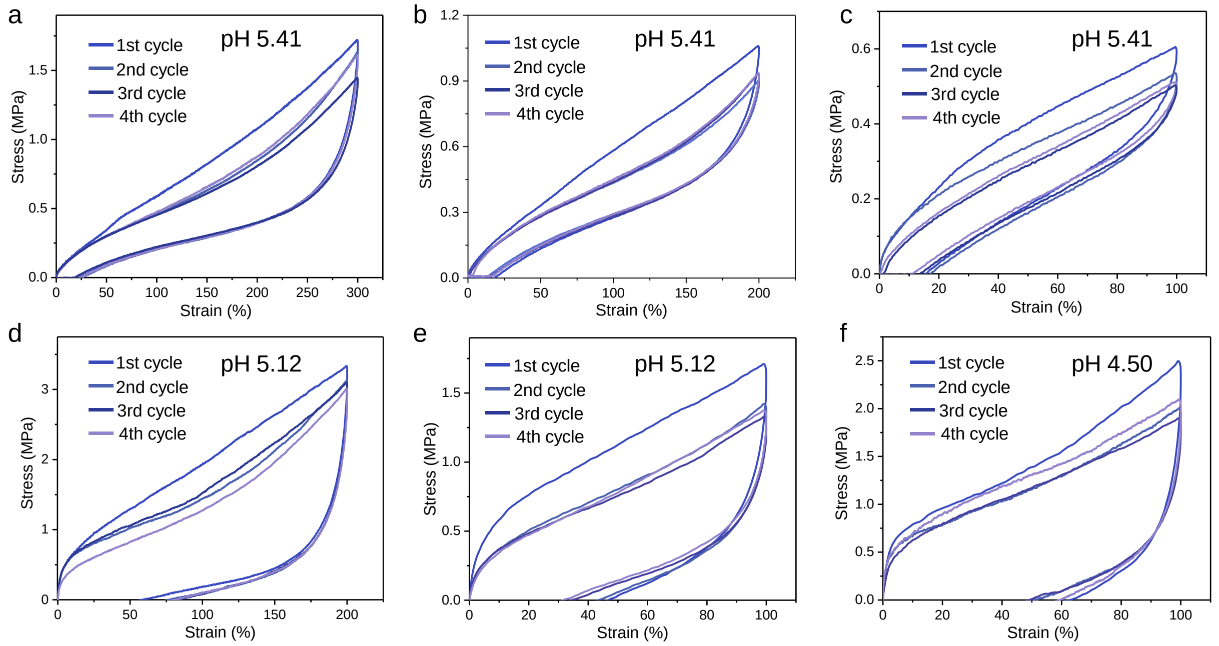

Supplementary Figure 23. Anelastic properties of the hydrogel microfibers of pH 5.41, pH 5.12 and pH 4.50. (**a**) Cyclic loadings of the pH 5.41 hydrogel microfiber to 300%, (**b**) 200% and (**c**) 100% strain (waiting time: 1 hour for each cycle). (**d**) Cyclic loadings of the pH 5.12 hydrogel microfiber to 200% and (**e**) 100% strain (waiting time: 1 hour for each cycle). (**f**) Cyclic loadings of the pH 4.50 hydrogel microfiber to 100% strain (waiting time: 2 hours for each cycle).

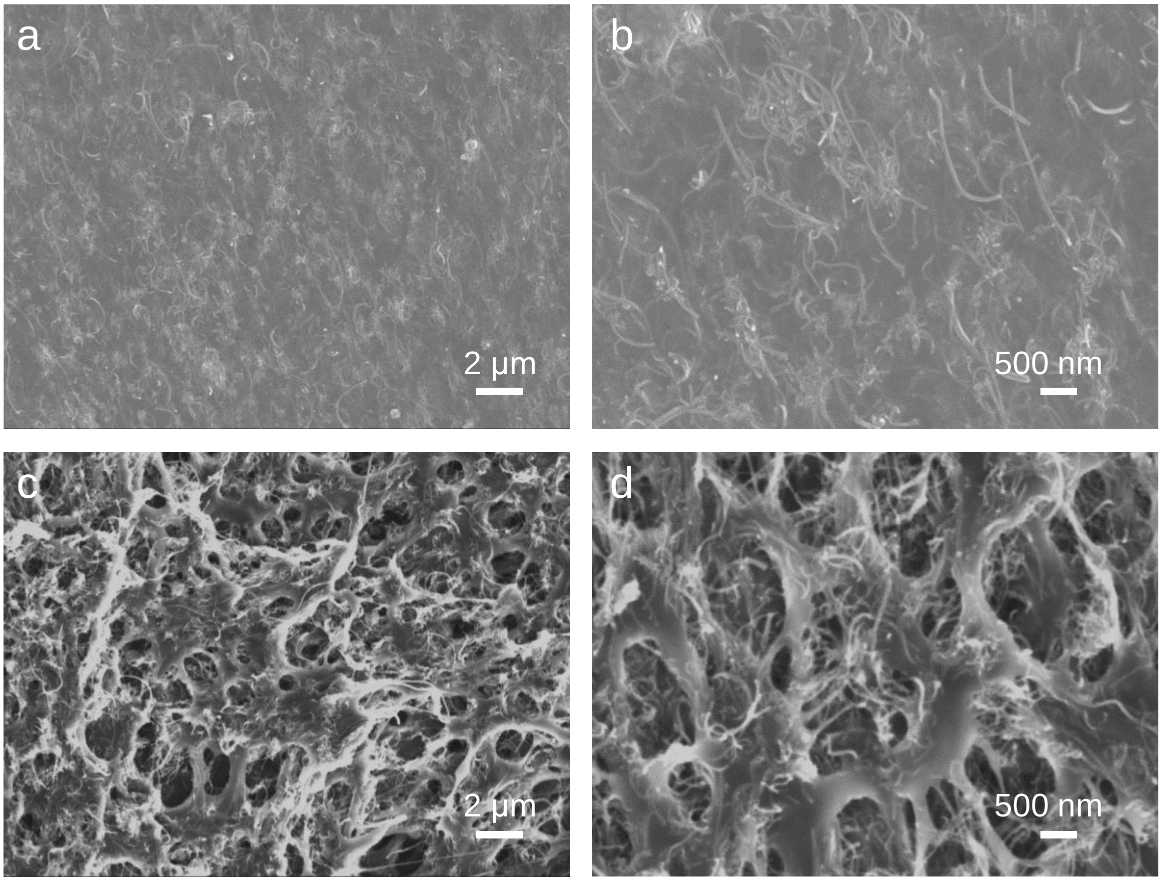

Supplementary Figure 24. (a,b) SEM images showing the surface and (c,d) internal structure of the pH 13.34 composite hydrogel (20 wt% SWCNTs).

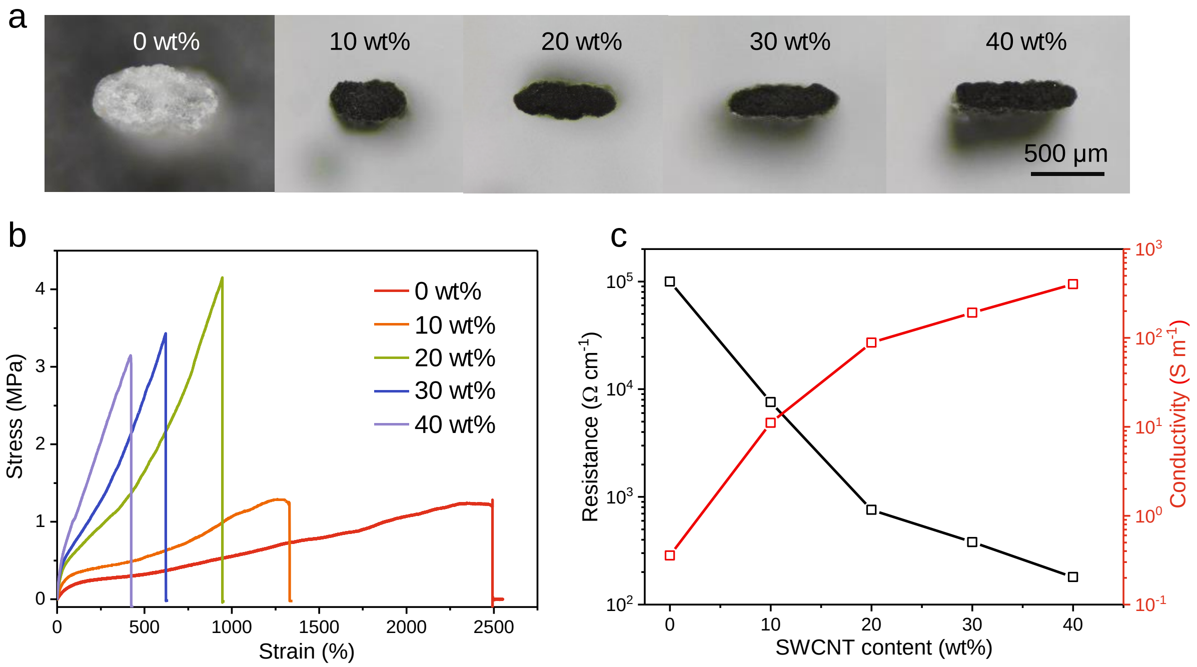

Supplementary Figure 25. Mechanical and electrical property of the pH 13.34 hydrogel microfibers modified by SWCNTs. (a) Cross-sectional optical microscope images of the pH 13.34 composite hydrogel with different contents of SWCNTs. (**b)** Strain-stress curves of the composite hydrogel fibers. (**c)** Resistance and conductivity of the composite hydrogel fibers.

As SWCNT content increases from 0 to 40 wt%, the breaking strain of the pH 13.34 composite hydrogel decreases from ~2500% to ~420%, alongside with the conductivity increasing from 0.4 S m^{-1} to 400.6 S m^{-1}.

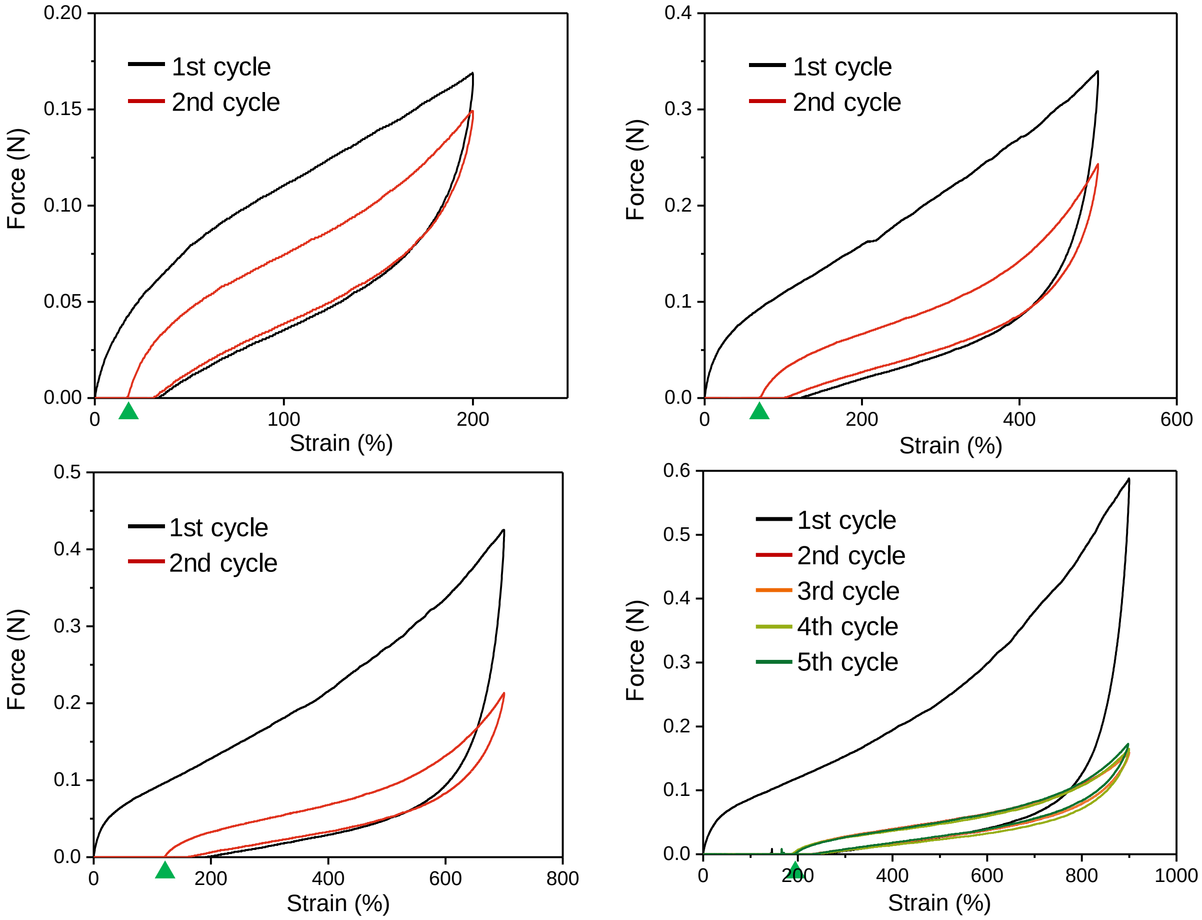

Supplementary Figure 26. Cyclic loadings of the pH 13.34 composite hydrogel (20 wt% SWCNTs) showing the permanent set produced by different prestrains.

The permanent set (permanent deformation) generated by 200%, 500%, 700% and 900% prestrain are around 17%, 70%, 121% and 192%, respectively.

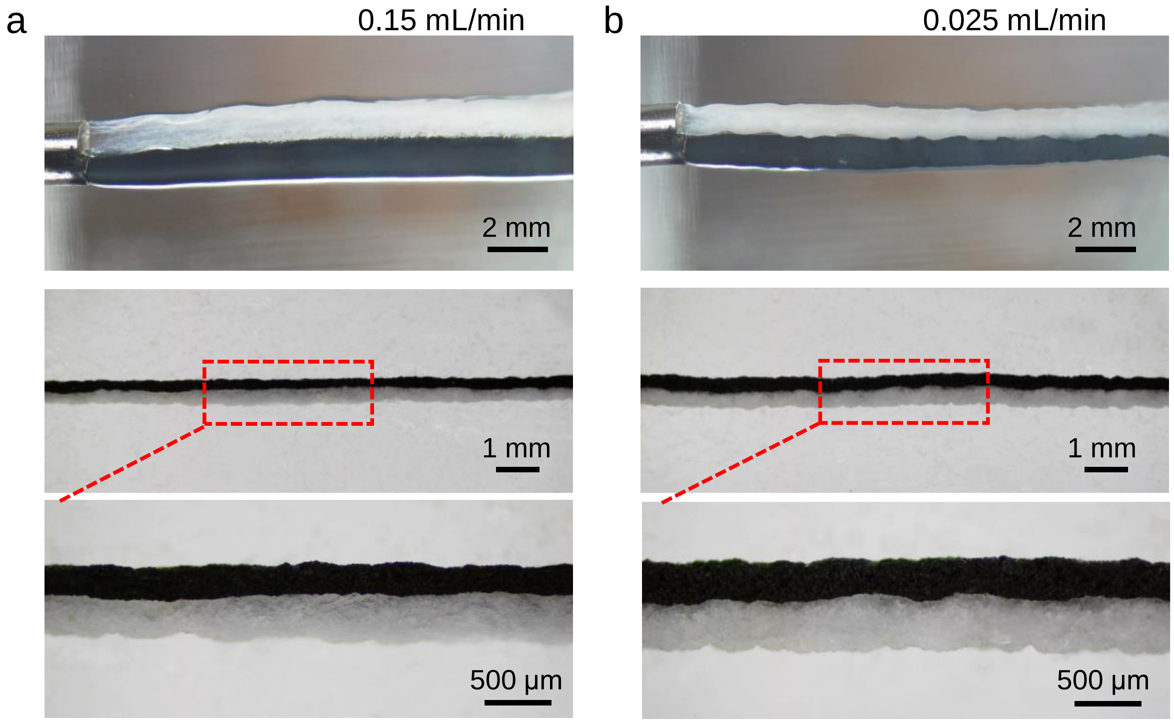

Supplementary Figure 27. (a) Formation of well-bonded interfaces in the Janus hydrogel fibers at flow rates of 0.15 mL/min and (b) 0.025 mL/min.

Upon extrusion into the methanol bath, the two phases were subjected to antisolvent phase separation induced dehydration process. In the beginning, we observed that the interface partially bonded with gaps, and the water in the two hydrogel phases came out from around the Janus structure. With the progress of phase separation, the Janus structure continuously shrank, and the two hydrogel dopes at the interface were forced to fully contact and bond together in the following dehydration process. The complete process was recorded by the microscope at a low flow rate of 0.025 mL/min as shown in the Supplementary Movie 6.

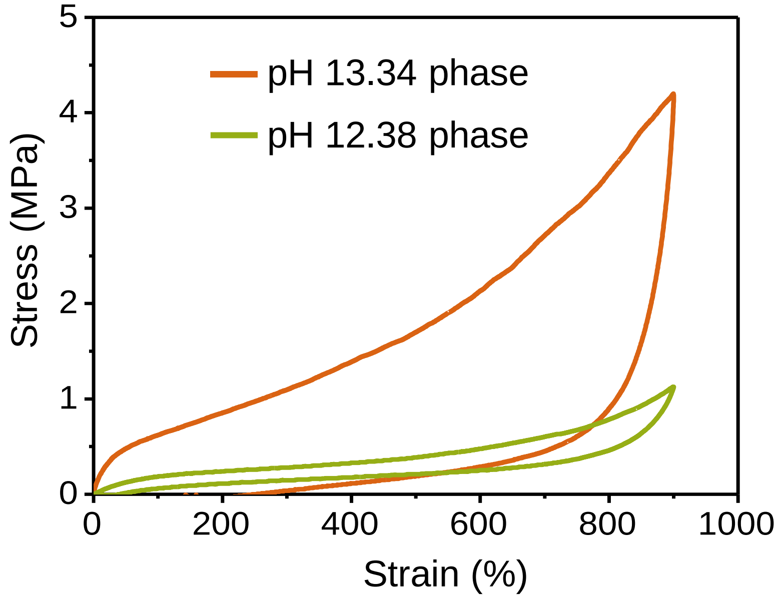

Supplementary Figure 28. Cyclic loading of the pH 12.38 microfiber and pH 13.34 composite microfiber to 900% strain.

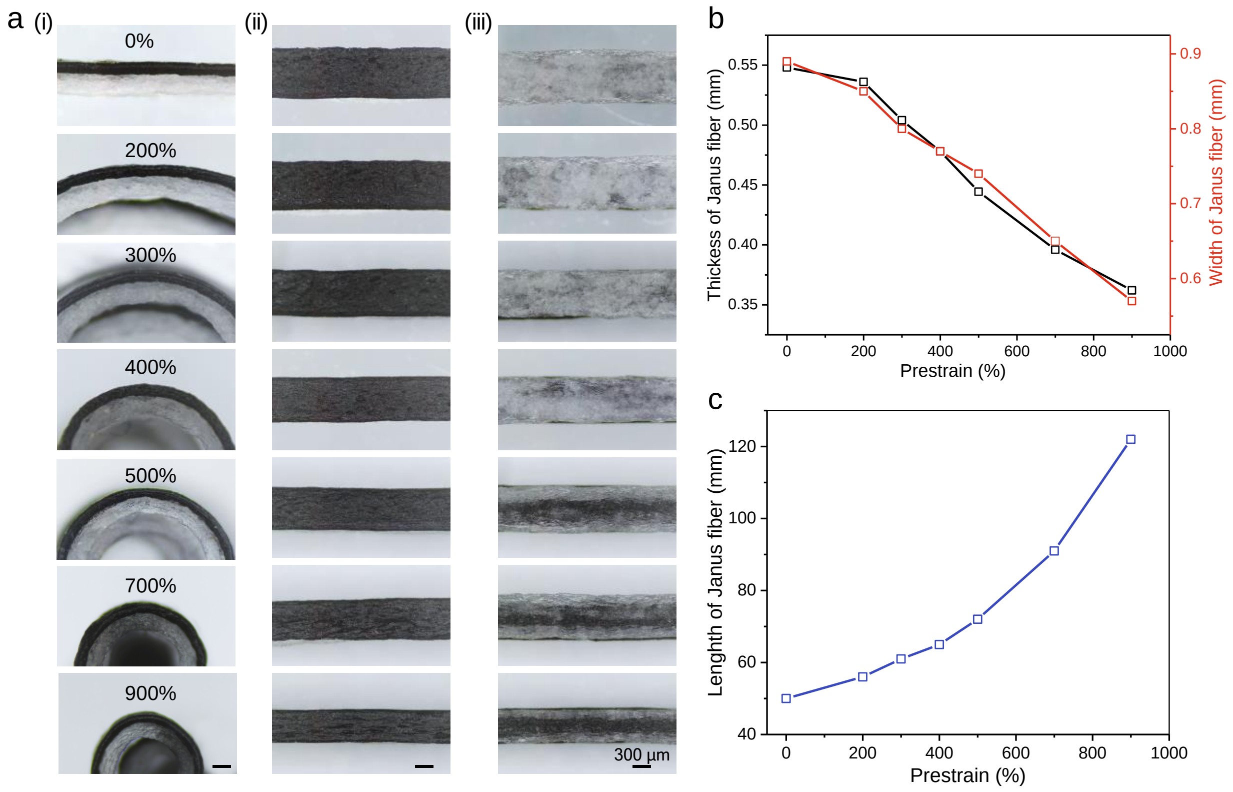

Supplementary Figure 29. Dimensional change of the Janus fibers released from different prestrains. (a) (i) Optical microscope images of the Janus fibers in the thickness direction, (ii) the pH 13.34 composite hydrogel phase and (iii) the pH 12.38 hydrogel phase in the width direction. **(b)** Thickness and width of the Janus hydrogel fiber as a function of the applied prestrain. **(c)** Final length of the Janus fiber as a function of the applied prestrain.

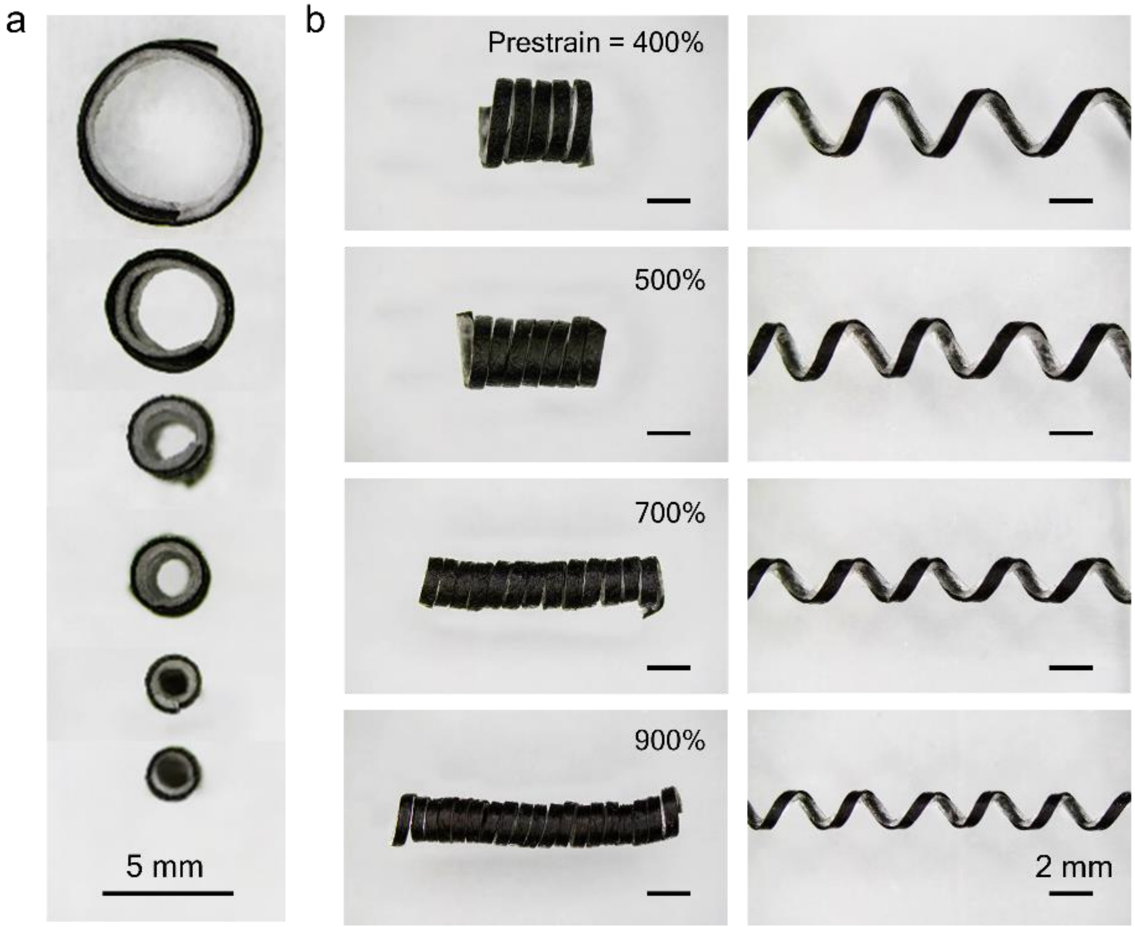

Supplementary Figure 30. Programmed helical hydrogel fibers with different diameters by applying different prestrains. (a) Cross-sectional optical microscope images of helical hydrogel fibers. (**b)** Optical microscope images of the helical fibers along the length direction in released states of 0% strain (left) and stretched states of 300% strain (right).

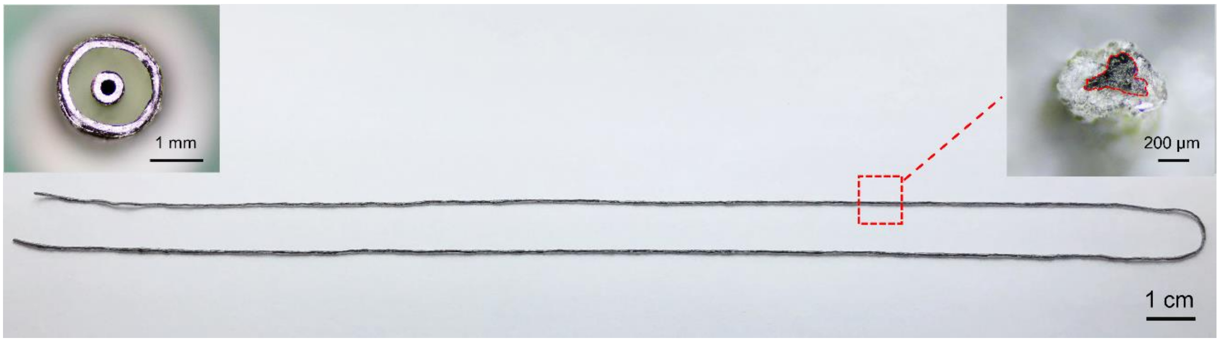

Supplementary Figure 31. Core-shell hydrogel microfiber. Inset microscope images show the coaxial spinneret and the core-shell structure.

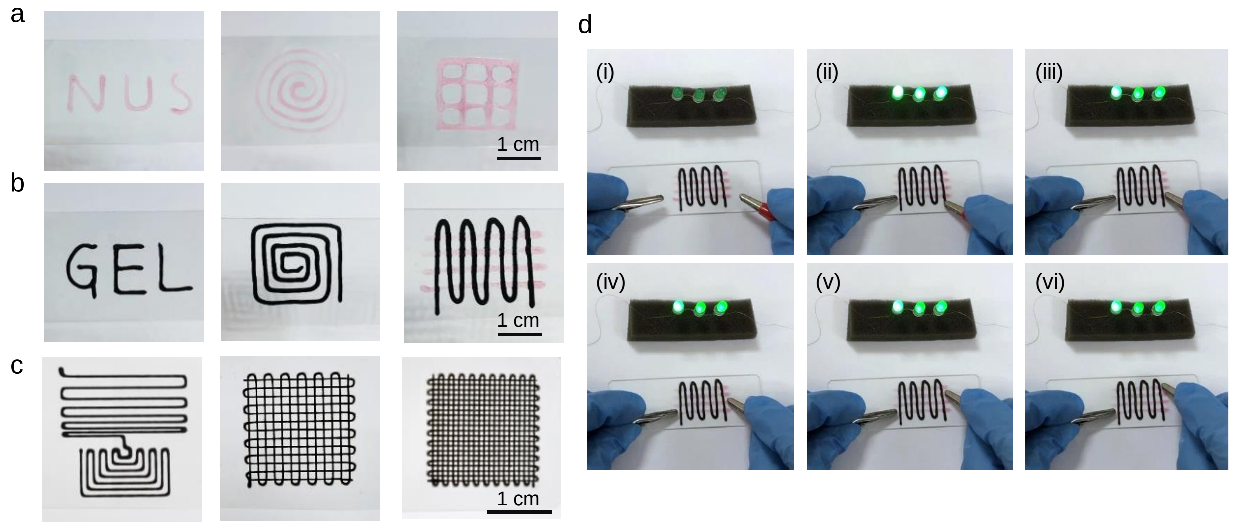

Supplementary Figure 32. Direct ink writing of hydrogel patterns and conductive hydrogel circuits. (a) Direct free writing of dyed hydrogel (pH 12.38) and **(b)** composite hydrogel patterns (pH 13.34) on glass slides. (**c)** Direct-ink-writing via 3D printing of composite hydrogel patterns (pH 13.34). (**d)** LED lighting via different conductive paths in the composite hydrogel patterns.

The hydrogel patterns and circuits were produced by hydrogel ink writing on the glass slides followed by sequential steps of soaking in methanol for 5 minutes and stabilization in the ambient air. The dyed pH 12.38 hydrogel and pH 13.34 composite hydrogel tightly bonded at the joints of the hydrogel pattern, and thus the hydrogel patterns were able to provide various conductive paths to lighten LED.

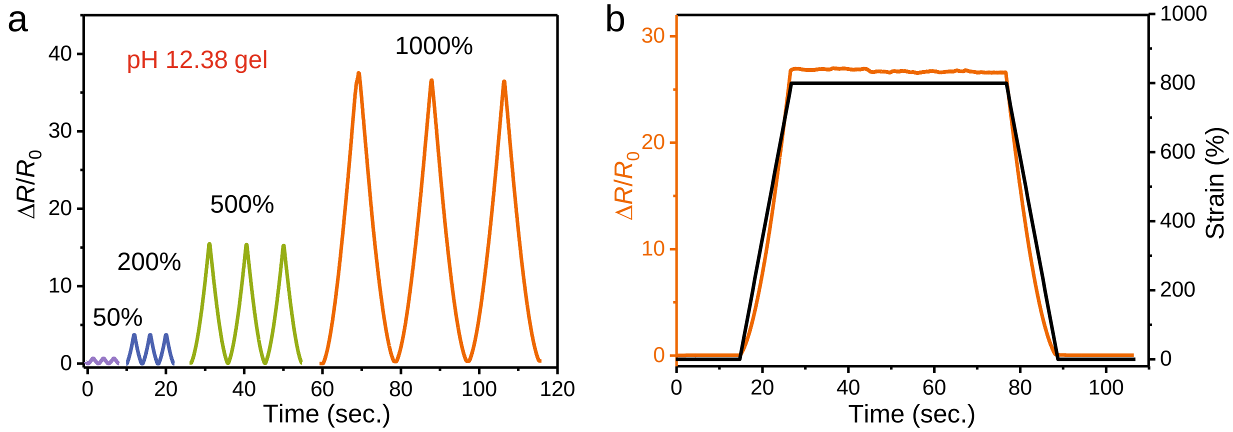

Supplementary Figure 33. Strain sensing of the highly stretchable, resilient pH 12.38 hydrogel fiber in a slow motion mode. (**a**) Resistance change under various cyclic strains. (**b**) Resistance change of the hydrogel fiber maintained at 800% strain for 50 seconds.

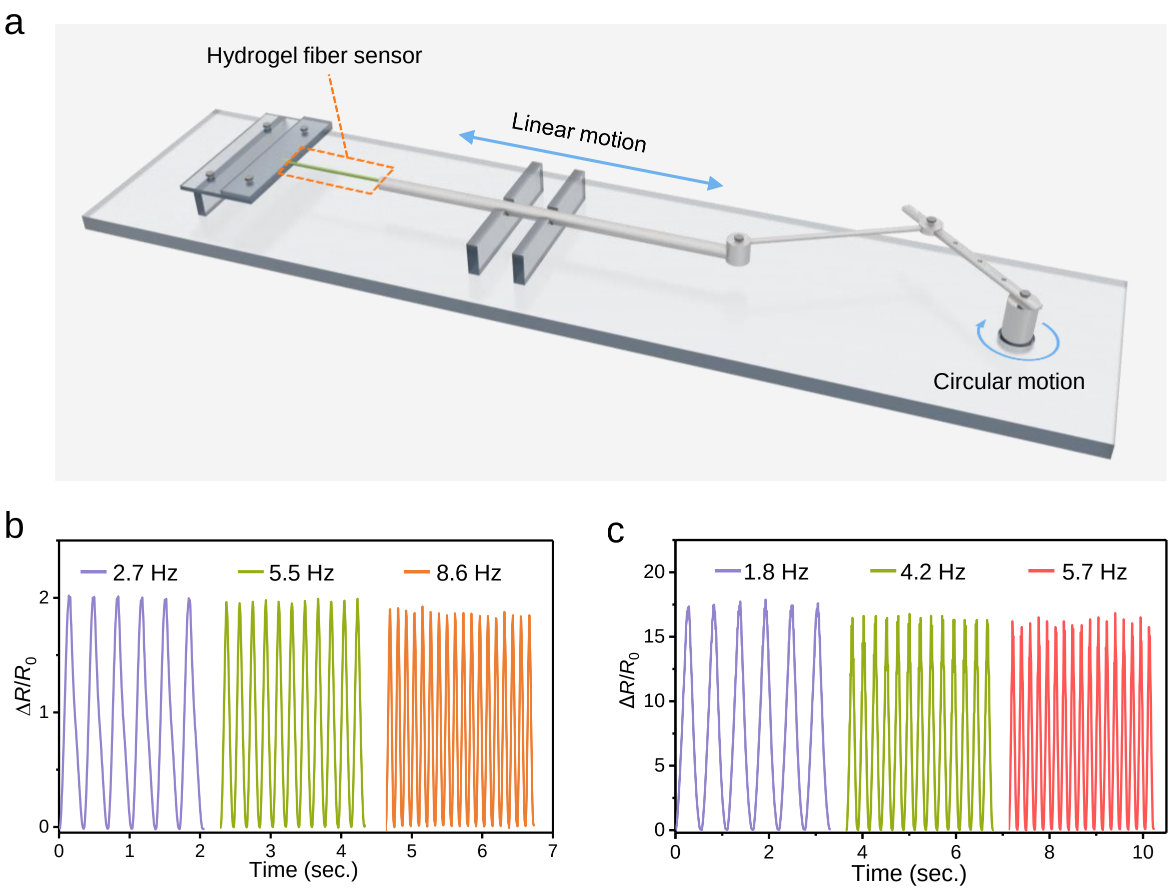

Supplementary Figure 34. High-frequency, large-strain sensing of the pH 12.38 hydrogel fiber in the fast-dynamic mechanical environment. (a) A home-made setup used to generate cyclic, linear motions in high frequency. **(b)** Resistance change under cyclic 100% strain at different stretching frequencies. **(c)** Resistance change under cyclic 600% strain at different stretching frequencies.

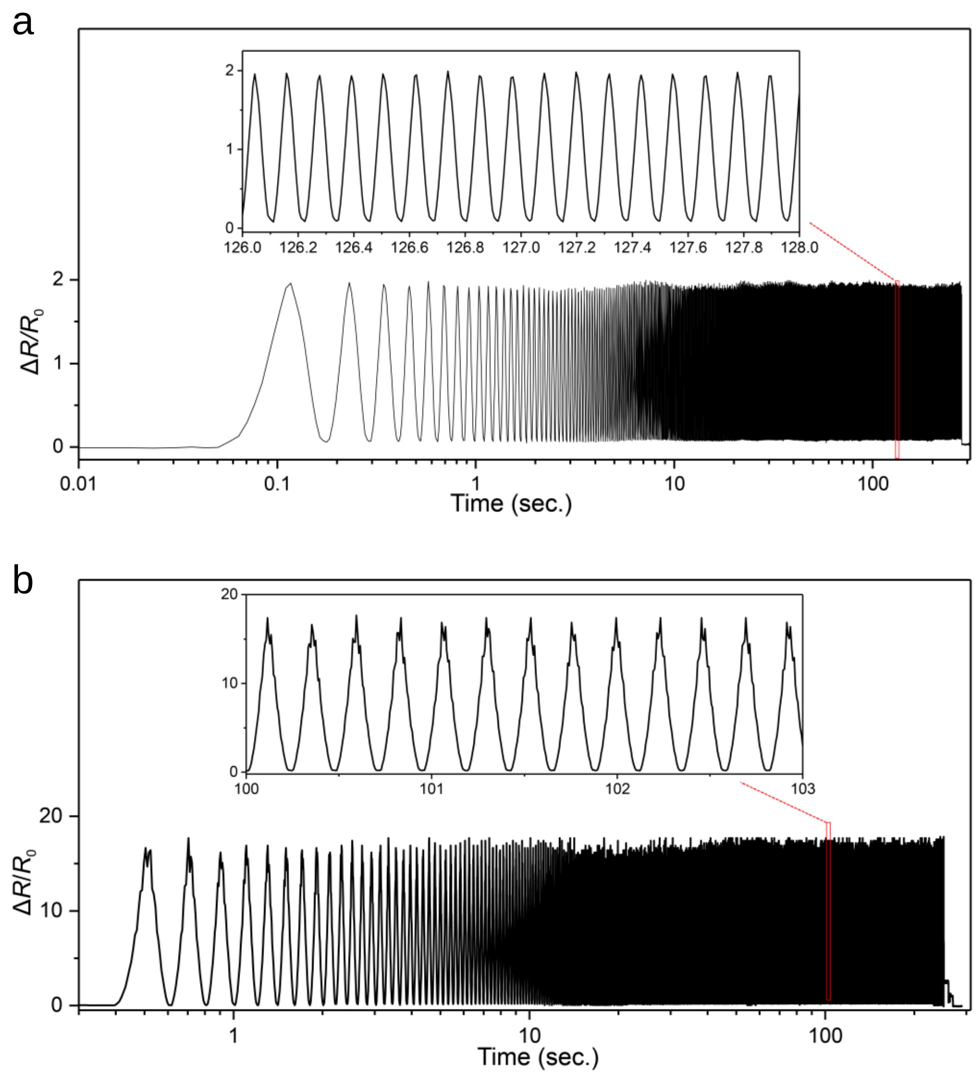

Supplementary Figure 35. Durability of the hydrogel strain sensors for monitoring high-frequency, large-strain motions. (**a**) Resistance change of the pH 12.38 hydrogel microfiber to cyclic 100% strain at 8.6 Hz for over 2000 cycles and (**b**) cyclic 600% strain at 4.2 Hz for over 1000 cycles.

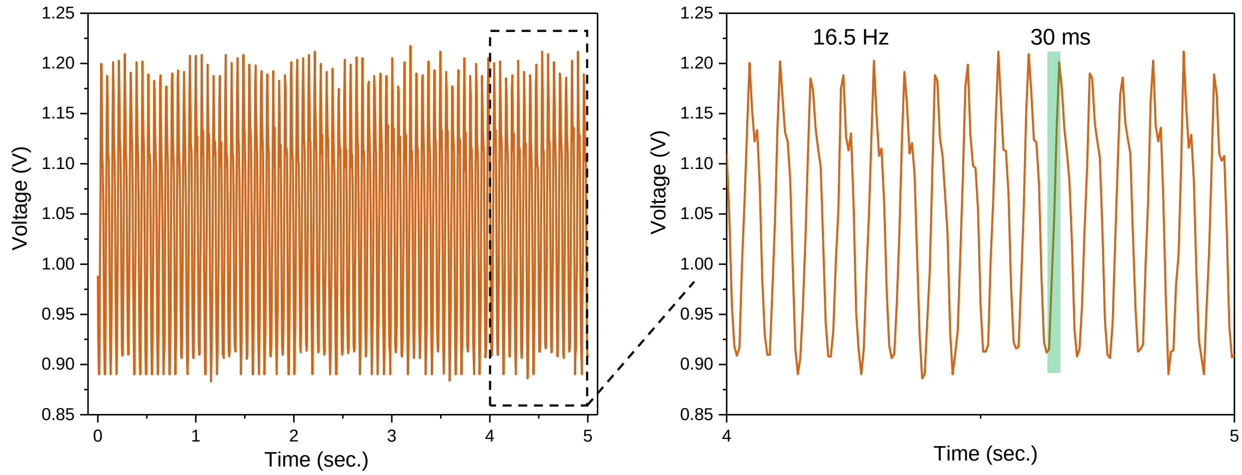

Supplementary Figure 36. Continuous sensing signals of wing flapping at ~16.5 Hz.

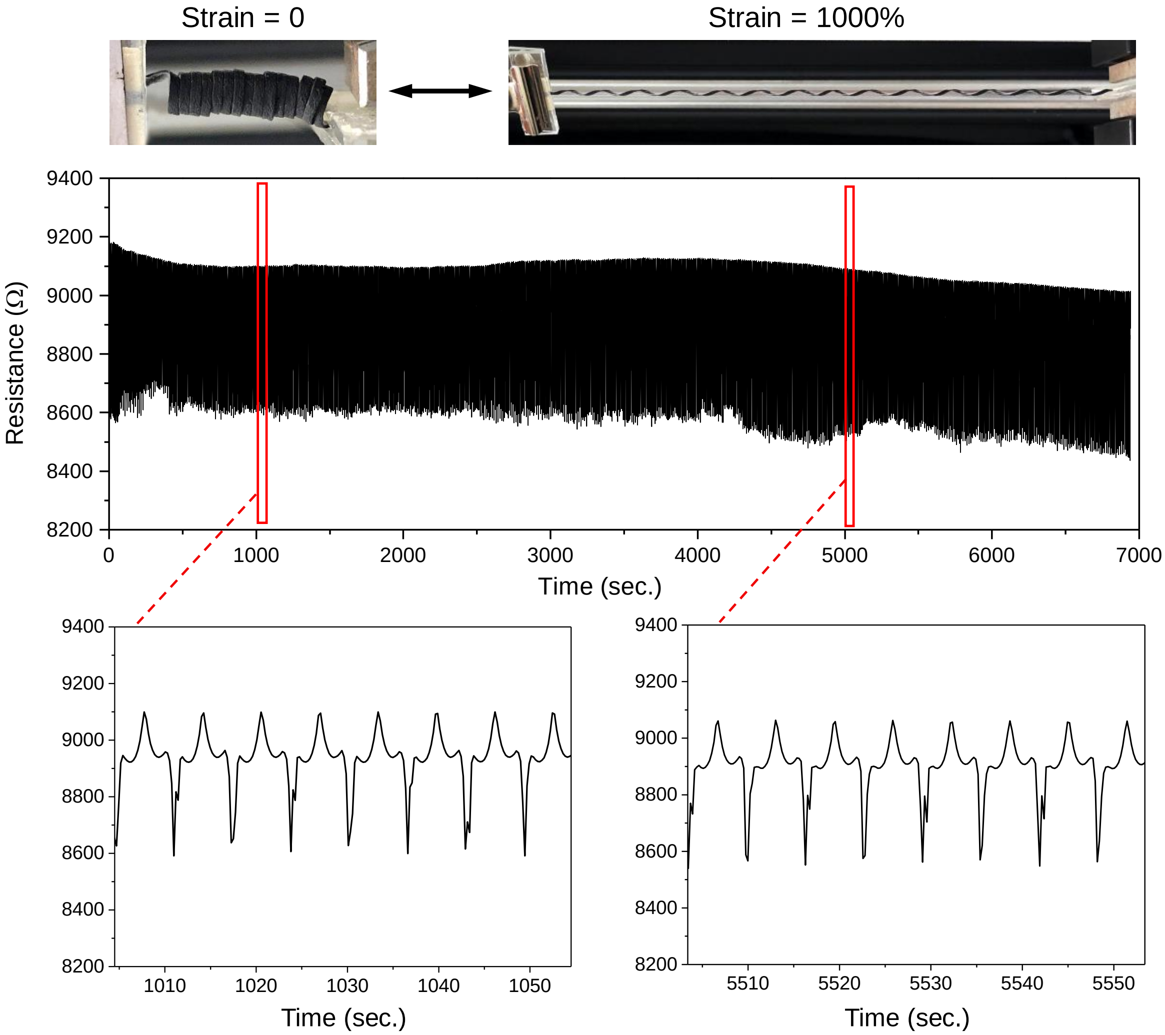

Supplementary Figure 37. Resistance change of the helical hydrogel conductor with a diameter of ~2.9 mm to cyclic 1000% strain for over 1000 cycles.

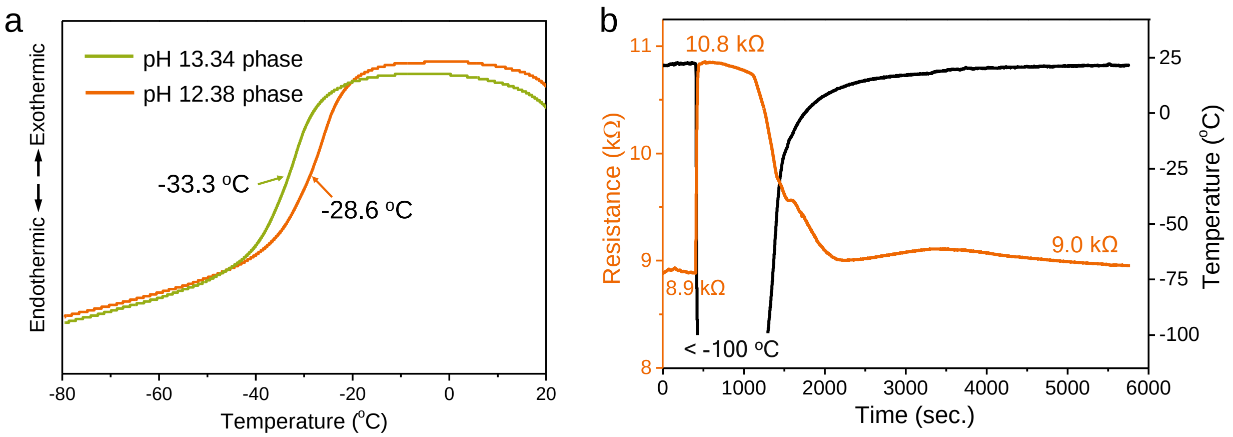

Supplementary Figure 38. (a) DSC curves of the pH 12.38 hydrogel microfiber and pH 13.34 composite hydrogel microfiber (20 wt% SWCNTs). (b) Temperature responsive resistance change as the hydrogel conductor was immersed into liquid nitrogen and recovery of the resistance as liquid nitrogen evaporated.

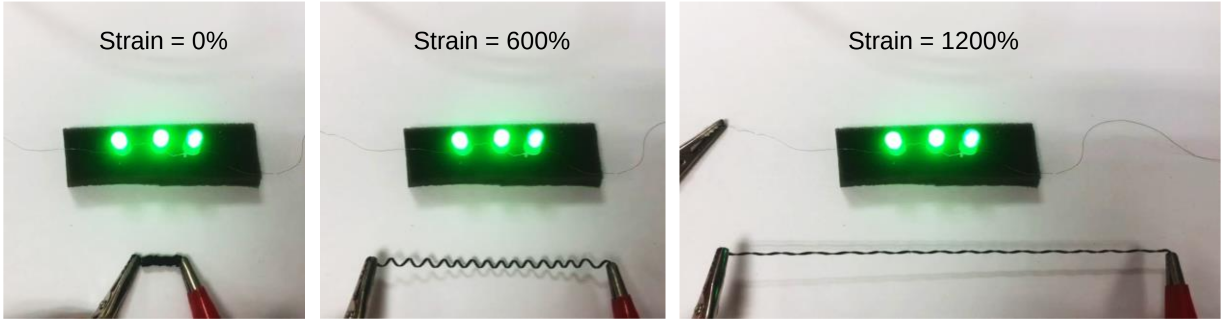

Supplementary Figure 39. Photographs of the LED lighting before and after stretching of the helical hydrogel conductor that was stored in the ambient air for 5 months.

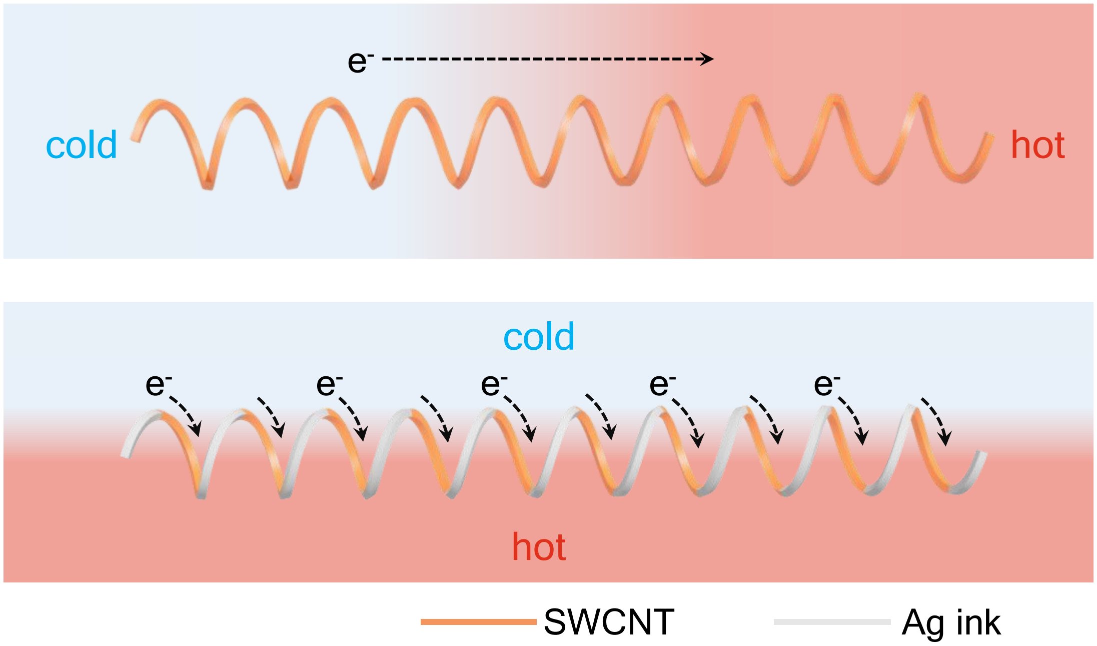

Supplementary Figure 40. Schematic illustration of the temperature gradient distribution on the TE hydrogel spring (top) and the Janus hydrogel spring consisting of TE coils electrically connected in series (bottom).

The helical hydrogel fiber exploits the Seebeck effect to harvest thermal energy from the temperature difference acted on the two ends of the helix, while the Janus hydrogel spring with half of each helical coil coated with Ag ink utilizes soft TE segments that are electrically connected in series for amplified power output.

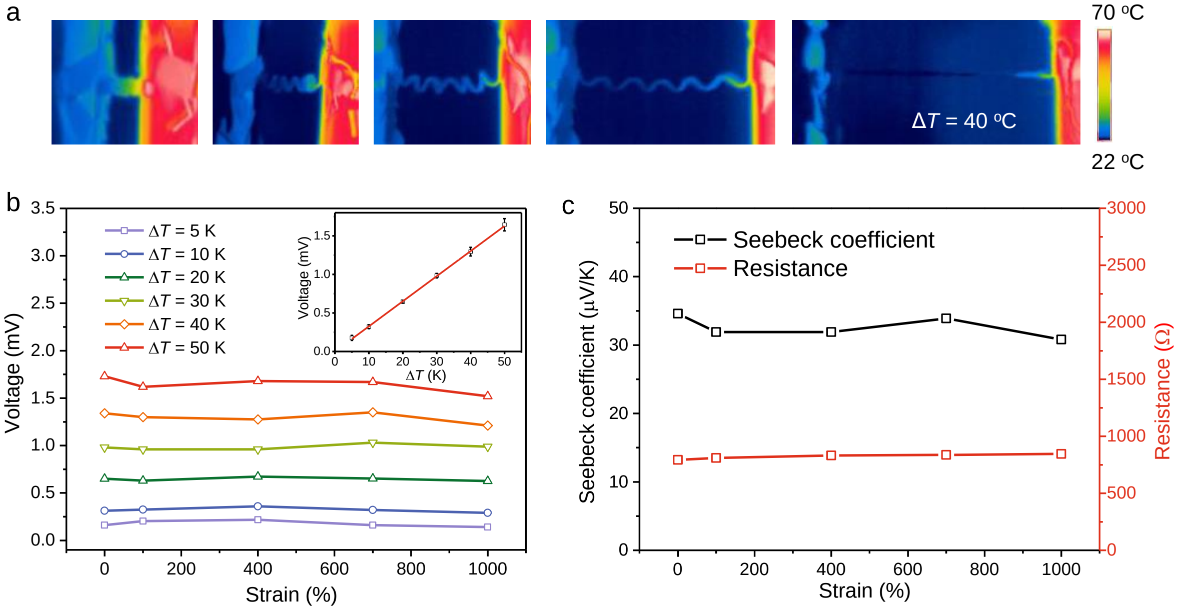

Supplementary Figure 41. TE performance of the ultrastretchable hydrogel spring. (a) Infrared images of a hydrogel spring with a diameter of ~3 mm at 0% , 100%, 400%, 700% and 1000% strain (from left to right) under a temperature difference of 40 ºC between the two ends. (**b)** Open-circuit voltages of the hydrogel spring at different strains and temperature differences. The inset figure shows the open-circuit voltage as a function of the temperature difference. Error bars represent SD. (**c)** The calculated Seebeck coefficient and resistance of the hydrogel spring at different strains.

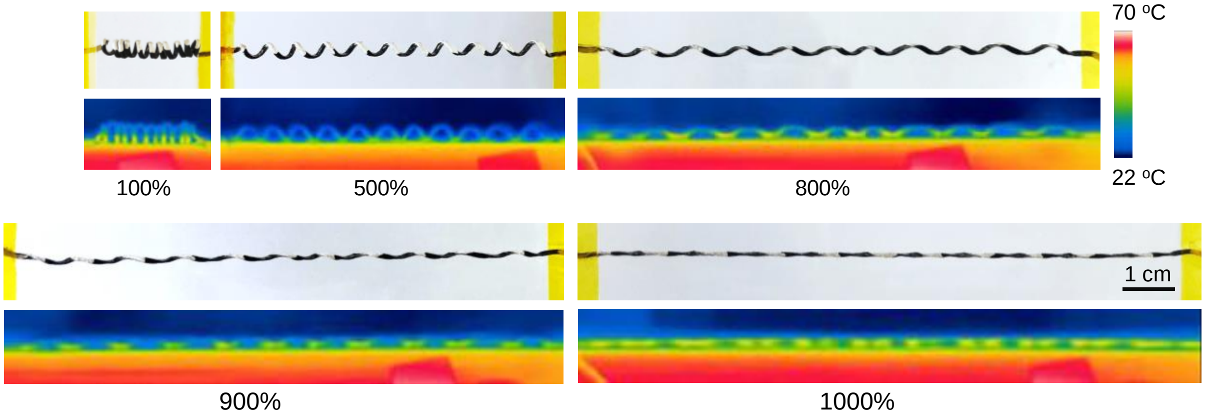

Supplementary Figure 42. Photographs and infrared images of the Janus hydrogel spring at different strains under the temperature difference of 40 °C between the hot substrate and the ambient air.

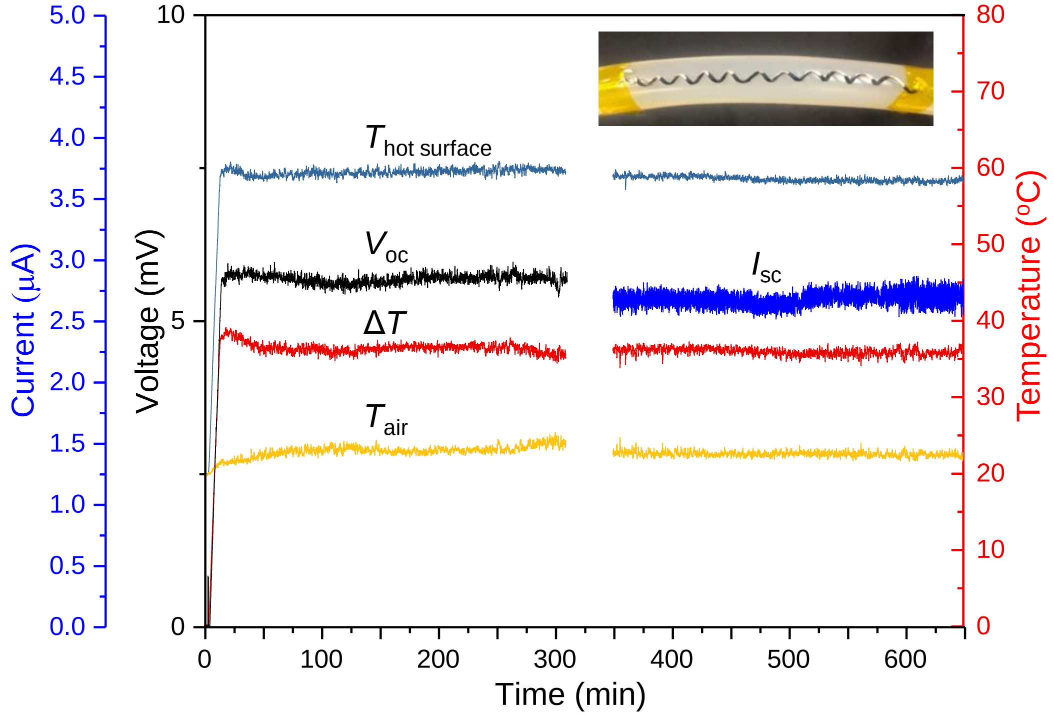

Supplementary Figure 43. V_{oc} and I_{sc} generated by the 500% strain Janus spring on the hot tube filled with circulating water of ~90 °C. The generated V_{oc} and I_{sc} were separately monitored for over 5 hours, and the gap interrupt in the figure was caused by changing the connection of electrodes for measurement of I_{sc}.

The surface temperature of the hot tube was ~60 °C, and the Janus spring consisting of 11 TE coils was exposed to a hot substrate-air temperature difference (ΔT) of ~36 °C. The generated V_{oc} of ~5.7 mV and I_{sc} of ~2.6 μA were stable over 5 hours.

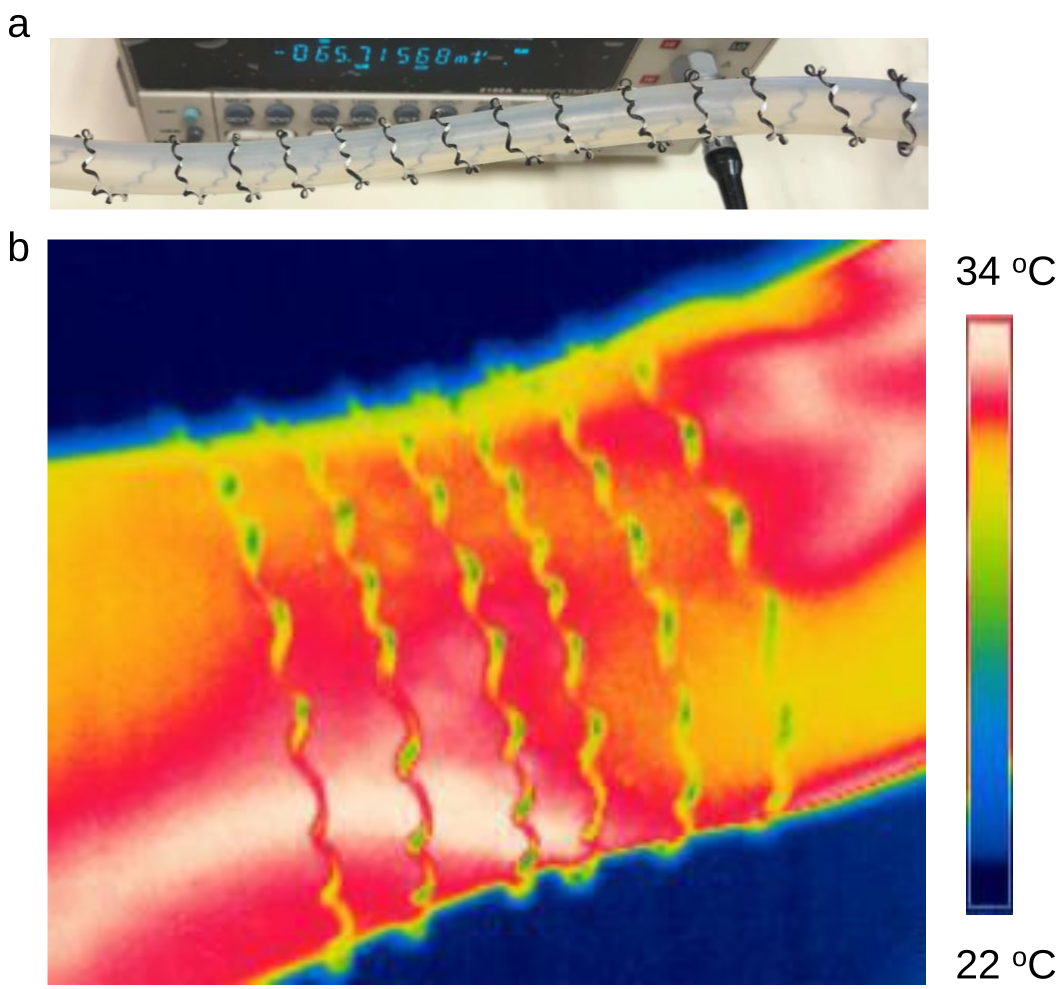

Supplementary Figure 44. (a) Photograph of the long Janus hydrogel spring wrapped around a hot tube filled with circulating water of ~90 °C and (b) an infrared image of the wearable Janus hydrogel spring bracelet.

Supplementary Table 1. Mechanical properties of the hydrogel microfibers of different pH. Data are presented as average value with standard deviation.

	Breaking strain (%)	Breaking stress (MPa)	Elastic modulus (MPa)	Toughness (MJ m^{-3})
pH 3.95	5 ± 2	47 ± 5	2050 ± 370	1.7 ± 1.1
pH 5.12	390 ± 60	6.7 ± 0.5	32.8 ± 3.6	15.4 ± 3.3
pH 6.35	850 ± 40	1.26 ± 0.07	0.28 ± 0.02	3.6 ± 0.1
pH 9.14	1090 ± 50	1.25 ± 0.07	0.24 ± 0.03	4.8 ± 0.3
pH 12.38	1540 ± 120	1.22 ± 0.04	0.27 ± 0.03	9.7 ± 1.5
pH 13.34	2530 ± 120	1.21 ± 0.12	0.34 ± 0.03	17.8 ± 1.6
pH 13.97	590 ± 80	2.5 ± 0.2	23.3 ± 6.5	11.9 ± 1.8

References

1. Cui, K. et al. Multiscale energy dissipation mechanism in tough and self-healing hydrogels. *Phys. Rev. Lett.* **121**, 185501 (2018).
2. Kongkhlang, T., Tashiro, K., Kotaki, M. & Chirachanchai, S. Electrospinning as a new technique to control the crystal morphology and molecular orientation of polyoxymethylene nanofibers. *J. Am. Chem. Soc.* **130**, 15460-15466 (2008).
