# Supplementary material for: Macromolecule conformational shaping for extreme mechanical programming of polymorphic hydrogel fibers
Source: Nat Commun. 2022 Jun 11;13:3369. doi: 10.1038/s41467-022-31047-3 (PMC9188594; doi:10.1038/s41467-022-31047-3)
Supplement: Supplementary file 3 — Description of Additional Supplementary Files [file 41467_2022_31047_MOESM3_ESM.pdf]

Description of Additional Supplementary Files

File Name: Supplementary Movie 1
Description: Continuous process of hydrogel filamentation and collection of fibers.

File Name: Supplementary Movie 2
Description: Filamentation of polyelectrolyte hydrogels of different pH in the methanol bath.

File Name: Supplementary Movie 3
Description: Formation of hydrogel ribbons using the spinning dope of pH 12.38.

File Name: Supplementary Movie 4
Description: Anelastic, elastic and plastic deformation of the hydrogel fibers of different pH.

File Name: Supplementary Movie 5
Description: Fabrication of Janus hydrogel fibers via the parallel-axial dualspinneret system.

File Name: Supplementary Movie 6
Description: Formation of bonded interfaces in the Janus fibers at different flow rates.

File Name: Supplementary Movie 7
Description: Formation of helical Janus fibers via the cold drawing process.

File Name: Supplementary Movie 8
Description: Highfrequency sensing of 100% and 600% strain.

File Name: Supplementary Movie 9
Description: Wireless monitoring of wing flapping motions.

File Name: Supplementary Movie 10
Description: Ultrastretchable and extremely low temperature tolerable LED lighting.
